# Metasurface–TMDCs for Strong Coupling and Emerging Nanoscale Light Sources

**DOI:** 10.3390/nano16211373

**Published:** 2026-10-27

**Authors:** Andergachew Mekonnen Berhe, Hao Wu, Haroldo T. Hattori, Andrey E. Miroshnichenko

**Affiliations:** School of Engineering and Technology, University of New South Wales at Canberra, Northcott Drive, Canberra, ACT 2610, Australia; a.berhe@unsw.edu.au (A.M.B.); hao.wu25@unsw.edu.au (H.W.); h.hattori@unsw.edu.au (H.T.H.)

**Keywords:** metasurface, TMDC monolayers, excitons, strong coupling, exciton–polariton, single-photon emission, nanoscale lasing, metalens

## Abstract

Metasurface–transition metal dichalcogenide (TMDC) monolayer hybrids are a promising platform for strong coupling and compact quantum nanophotonic devices. In this review, we discuss the main theories, design approaches, recent experiments, and applications of these systems, focusing on single-photon emission and lasing. We explain how the mode volume and quality factor work together in these systems. Dielectric and plasmonic metasurfaces provide different balances of optical loss and field confinement. Because the reported systems differ in TMDC material, temperature, exciton linewidth, and field overlap, their Rabi splittings alone cannot establish an intrinsic ranking of the two platforms. In the strong coupling regime, polariton nonlinearities could enable photon blockade and high-purity single-photon emission, but achieving this at room temperature is still mostly theoretical. Photon lasing has been shown in metasurface–TMDC systems, but clear evidence of polariton lasing from a TMDC monolayer combined with dielectric or plasmonic metasurfaces is still missing. The main challenges include exciton–exciton annihilation and weak mode overlap in dielectric structures, as well as very short photon lifetimes in metallic resonators. Finally, we discuss experimental benchmarks and the scalable cleanroom fabrication of high-*Q* plasmonic metasurfaces, including dielectric metasurfaces. These are applicable to room-temperature, directional, ultrafast, and energy-efficient nanoscale light sources. Furthermore, low-loss dielectric metasurfaces are summarised for manipulating the generated emitted light, which is crucial for directing and manipulating the light on-chip and for directly collimating and aligning it with an optical fibre.

## 1. Introduction

Light–matter interaction is essential for many photonic technologies, such as lasers, nonlinear optical devices, quantum light sources, and integrated optoelectronic systems. At the nanoscale, though, the interaction between an optical field and an active material is limited by how much they overlap in space and time. This creates a key challenge in making nanoscale light emitters smaller and more energy-efficient [1]. This limitation becomes particularly important for atomically thin semiconductors: a TMDC monolayer is only approximately 0.7 nm thick, confining the active excitonic material to an extremely small physical volume while retaining strong optical transitions [2,3]. Efficient light–matter interaction therefore requires not only a nanoscale active medium but also an optical mode whose electromagnetic energy is confined to a comparably small region and strongly overlaps the emitter. A central objective of nanophotonics is consequently to engineer resonant structures that simultaneously provide strong spatial confinement, characterised by a small effective optical mode volume $V_{\mathrm{eff}}$, and sufficient temporal confinement of the electromagnetic field [4]. Reducing $V_{\mathrm{eff}}$ increases the electromagnetic field per photon and the local optical density of states at the emitter position. This enhancement promotes spontaneous emission and emitter–cavity coupling and, when the interaction is sufficiently strong, enables coherent energy exchange between light and matter [2,5,6]. Resonant metasurfaces provide a versatile means of matching the nanoscale optical mode to the atomically thin active medium, since their response can be adjusted via the material composition, geometry, symmetry, periodicity, and near-field distribution. These degrees of freedom enable the optical field to be concentrated and oriented at the physical position of the TMDC monolayer, providing simultaneous control over resonant confinement, field–emitter overlap, and ultimately the strength of light–matter interaction [4,7].

The performance of a resonant metasurface is governed by the balance between temporal and spatial light confinement, characterised by the quality factor *Q* and effective mode volume $V_{\mathrm{eff}}$, respectively. Thus, the $Q/V_{\mathrm{eff}}$ provides an important figure of merit for enhanced light–matter interaction and spontaneous emission control [8]. Dielectric and plasmonic metasurfaces approach this balance through complementary physical mechanisms. High-index dielectric resonators exhibit low material absorption and can suppress radiative leakage through multipolar interference, Fano resonances, and bound states in the continuum (BIC), thereby supporting narrow resonances and high *Q* factors [7,9]. BIC and quasi-BIC resonances of dielectrics can be engineered within the visible spectral range of TMDC excitons and have enabled strong exciton–photon coupling [10]. Furthermore, BIC-based dielectric gratings have been investigated for enhanced refractive index sensing [11], while Fano-resonant hybrid metastructures combining dielectric and plasmonic components have demonstrated high spectral sensitivity [12] However, an ultrahigh *Q* does not necessarily maximise the interaction with an externally positioned monolayer, because the electromagnetic energy can remain distributed over a comparatively large volume or predominantly inside the dielectric resonator. As a result, plasmonic metasurfaces provide a complementary route by exploiting surface plasmon polaritons (SPPs) and localised surface plasmon resonances (LSPRs) to concentrate electromagnetic energy far below the diffraction limit, producing intense near-fields and exceptionally small $V_{\mathrm{eff}}$ at metal–dielectric interfaces, edges, and nanogaps, albeit with a lower *Q* imposed primarily by ohmic dissipation [13]. Here, a periodic plasmonic array can further suppress radiative leakage through collective surface lattice and BIC-related resonances while retaining strong nanoscale near-field confinement. These complementary confinement mechanisms become particularly important when the resonator is hybridised with TMDC monolayers [14,15].

As a result, to achieve strong coupling with larger Rabi splitting in TMDC monolayers, the attainable coupling strength is therefore determined not by *Q* alone but by the combined effects of $V_{\mathrm{eff}}$, the excitonic oscillator strength, spectral detuning, and the spatial and orientational overlap between the resonant field and the excitonic transition dipole. This latter requirement is particularly important because the bright excitonic transitions of monolayer TMDCs are predominantly in-plane [2]. Consequently, the central resonator design problem in this work is to determine whether the long photon lifetime provided by high-*Q* dielectric resonances or the deep-subwavelength field confinement provided by plasmonic resonances offers the more effective route to strong coupling with an atomically thin semiconductor.

**Figure 1:**
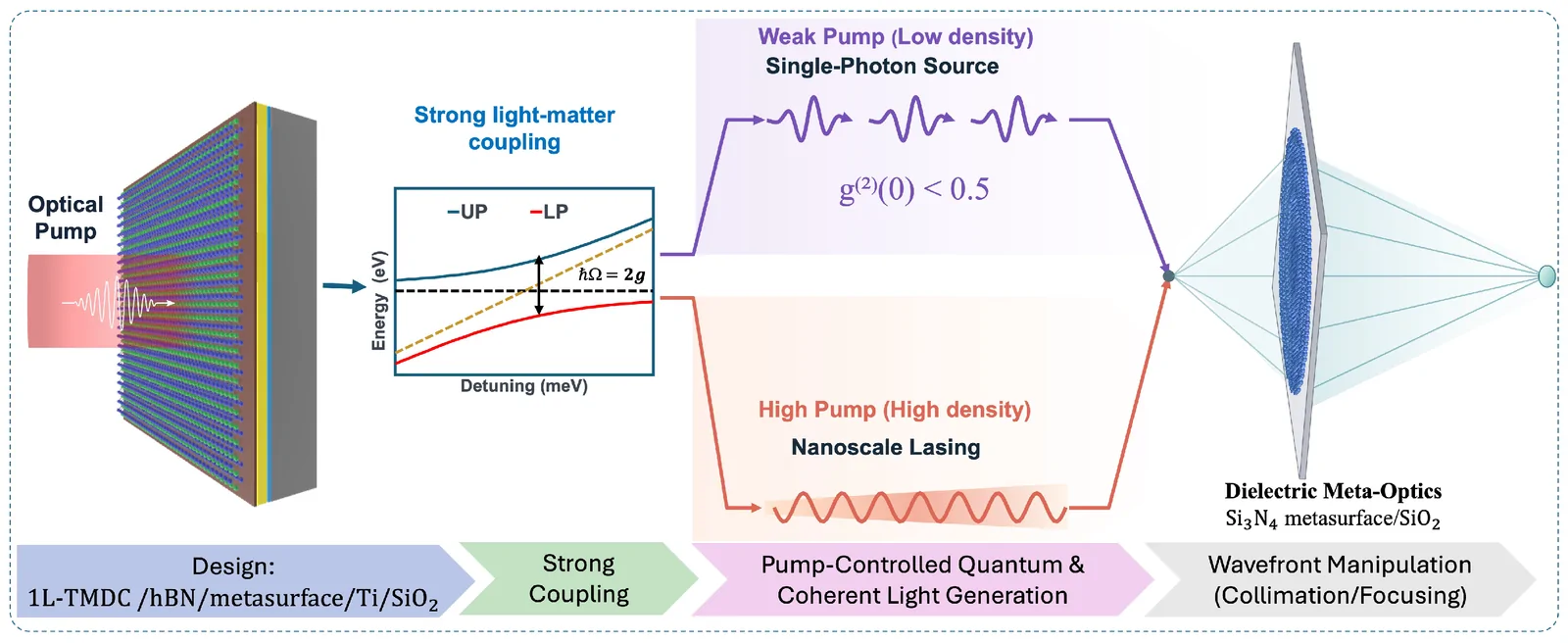
Conceptual schematic showing the proposed integrated TMDC and metasurface platform. It illustrates how optical pumping of the TMDC monolayer–metasurface hybrid leads to strong exciton–plasmon or photon coupling (see Section 6 for the metasurface’s fabrication). When the excitation density is low, the system emits single photons. As the excitation density increases, the system moves toward lasing. The system then collects and shapes the single photons or laser light using a ${\mathrm{Si}}_{3}$$N_{4}$ nanopillar-based metalens. Alternatively, other low-loss dielectric materials such as ${\mathrm{TiO}}_{2}$ can be considered. The collimated output facilitates efficient coupling into optical fibres or on-chip photonic waveguides.

Metasurface–TMDC hybrids support several distinct routes to nanoscale light generation, including Purcell-enhanced emission from localised quantum emitters, conventional photon lasing, and emission involving strongly coupled exciton–polaritons. One important direction is quantum emission, as monolayer ${\mathrm{WSe}}_{2}$ supports strain- and defect-localised excitonic states that act as single-photon emitters [16,17]. The deterministic positioning of such emitters within plasmonic nanocavities has yielded substantial enhancements in their spontaneous emission rate and photon extraction efficiency through strong nanoscale field confinement [5]. Strong exciton–photon coupling offers a complementary route in which the anharmonic energy spectrum of the hybrid system suppresses multiphoton occupation and produces antibunched light, provided that the polariton nonlinearity exceeds the relevant dissipation rates as required for photon blockade [18,19]. At moderate excitation densities, but below the Mott threshold at which excitons ionise and oscillator strength collapses, the same cavity–emitter architecture can instead be developed toward coherent nanoscale light sources. Monolayer TMDCs have already enabled ultracompact laser action at cryogenic temperatures when integrated with photonic crystal and whispering gallery microcavities [20,21]. At the same time, exciton–polariton condensation and lasing offer a different strong coupling pathway. In this case, stimulated scattering fills a polariton state with many particles but does not need the population inversion required for regular photon lasing [22]. As a result, a resonant metasurface is therefore treated as an engineered electromagnetic environment for generating, confining, and controlling quantum or coherent emission at the nanoscale, rather than merely as a passive spectral resonance [23,24]. A complementary low-loss dielectric metasurface can subsequently be positioned as an external wavefront control element, manipulating the generated radiation without itself participating in the light–matter coupling. By imposing a spatially varying phase profile through subwavelength dielectric meta-atoms, such a meta-optic can collect and focus the emitted light as a metalens [25], while superimposing an azimuthal phase ramp of integer topological charge enables the generation of a focused vortex beam carrying the corresponding orbital angular momentum per photon [26,27]. As can be seen in Figure 1, the two metasurface stages thus perform complementary functions within the overall platform: the first engineers the local density of optical states, the field confinement, and the exciton–photon interaction required for nanoscale light generation, whereas the second controls the collection, focusing, and spatial phase structure of the emitted field.

Accordingly, this work follows a unified design-to-application progression. Dielectric and plasmonic metasurfaces are first engineered independently to establish controlled optical resonances and to quantify the trade-off between the photon storage time and deep-subwavelength field confinement. These resonators are then hybridised with TMDC monolayers to investigate strong coupling and identify the architecture that best balances Rabi splitting and the polariton linewidth. The resulting hybrid platform is developed toward nanoscale light generation, with an emphasis on single-photon emission and lasing. Finally, a separate low-loss dielectric metasurface manipulates the generated radiation through metalens focusing and focused vortex beam formation. This sequence connects resonator engineering, exciton–photon hybridisation, light generation, and wavefront control within a single metasurface-based photonic framework.

## 2. Fundamentals of TMDC Monolayers: Band Structure, Excitons, and 2D Advantage

The family of 2D materials spans semimetals, semiconductors, insulators, superconductors, and magnets and now underpins a distinct branch of nanophotonics in which the optical response is carried by confined polaritons rather than by bulk permittivity alone [28,29]. This coverage, combined with dangling-bond-free surfaces that permit arbitrary vertical stacking, is what has made the layered family distinct and crucial in nanophotonics [30]. Graphene, black phosphorus, hexagonal boron nitride (hBN), and the TMDCs share an atomically thin geometry. Still, their electronic and optical responses vary fundamentally: each supports a different polaritonic species, over a different spectral window, with different loss characteristics. They should therefore not be treated as interchangeable active media for strong light–matter coupling in the visible and near-infrared (NIR) range [31,32].

It should be noted that other 2D materials also have distinct applications but are missing compared with TMDCs in the visible regime. For example, graphene offers an exceptional broadband response, nearly frequency-independent interband absorbance, and gate-tunable plasmons that confine light to deeply subwavelength volumes in the mid-infrared and terahertz regimes [33]. However, graphene lacks an intrinsic gap and a discrete visible exciton suitable for conventional exciton–polariton formation [34]. Black phosphorus also possesses a direct, thickness-tunable band gap and strongly anisotropic, polarisation-dependent excitonic absorption, making it an attractive platform for near- to mid-infrared photonics. However, its rapid degradation through oxygen-, moisture-, and light-assisted ambient oxidation remains a major challenge for reproducible device fabrication and stable cavity integration [35].

Hexagonal boron nitride (hBN) also plays an important role but is missing from the visible regime. Compared with TMDCs, it suppresses disorder and charge instabilities, narrowing the TMDC exciton linewidths toward the homogeneous limit. In addition, optically active point defects in hBN act as bright, stable, room-temperature single-photon emitters, making hBN an important quantum light source in its own right. Beyond quantum emission, hBN naturally supports low-loss hyperbolic phonon polaritons in its mid-infrared regime, enabling deep sub-diffractional optical confinement and enhanced light–matter interactions [36]. Its intrinsic band edge, however, is an indirect band gap of approximately 5.95 eV in the deep ultraviolet range, so hBN cannot substitute the bright visible and near-infrared excitons of TMDC monolayers. Instead, hBN complements TMDCs by providing a high-quality dielectric environment and hosting defect-based quantum emitters. At the same time, the TMDC monolayers supply the strong visible/NIR excitonic transitions required for efficient light–matter interaction (see Table 1 for further comparison).

Among these classes, TMDCs are characterised by the chemical formula ${\mathrm{MX}}_{2}$, where M is commonly a group-VI transition metal, such as Mo or W, and X is a chalcogen, such as S, Se, or Te. Other TMDCs include group-V (vanadium (V)-, niobium (Nb)-, and tantalum (Ta)-based) and group-X (palladium (Pd)- and platinum (Pt)-based) compounds. Group-V TMDCs are commonly metallic, whereas group-X TMDCs exhibit diverse electronic properties, including semiconducting and semimetallic behaviour [37]. However, semiconducting group-VI TMDCs are particularly suitable for visible and near-infrared (NIR) strong light–matter coupling, owing to their pronounced excitonic resonances and high oscillator strengths [38]. These single-layer semiconductors consist of a hexagonal plane of transition metal atoms confined between two chalcogen planes, forming an X–M–X trilayer with a physical thickness of approximately 0.6–0.8 nm. Strong covalent bonding within each layer and comparatively weak van der Waals attraction between neighbouring layers allow the crystals to be exfoliated or grown as atomically thin sheets while largely preserving their in-plane lattice structure [37,39].

**Table 1:** Comparison of representative two-dimensional (2D) materials for visible and NIR nanophotonics and light–matter coupling. The table summarises each material’s electronic structure, principal optical range, key advantages, and limitations for light–matter interaction. Band gaps and optical ranges are approximate and vary with the layer number, strain, dielectric environment, and sample quality.

Material	Electronic Structure	Principal Optical Range	Advantages	Limitations for Strong Coupling	Refs.
Graphene	Gapless semimetal; no intrinsic band gap in monolayers	Broadband (THz–visible); plasmonic response strongest in the THz and MIR	High carrier mobility, electrically tunable conductivity, broadband absorption	Gapless semimetal (no conventional discrete exciton), limiting conventional exciton–polariton formation	[34,40]
Black phosphorus	Direct, thickness-dependent band gap (∼0.3 eV in bulk to ∼1.5–2 eV in the monolayer)	Near- to mid-infrared, extending toward the visible for monolayers	Strong in-plane anisotropy, tunable direct band gap, and high carrier mobility	Rapid oxygen-, moisture-, and light-assisted degradation; anisotropy complicates reproducible cavity integration	[41]
hBN	Wide-band-gap indirect semiconductor ($E_{g}\approx 5.95$ eV)	Deep-ultraviolet band edge response; visible emission from defect centres	Chemically stable dielectric, excellent metal–TMDC spacer and host for quantum emitters	Not a visible/NIR excitonic semiconductor; visible emission originates from defect states rather than the band edge	[42,43]
1-L TMDCs	Direct optical transitions at the $K/K^{\prime }$ valleys in monolayers and pronounced excitonic resonances	Visible to NIR (∼1.1–2.1 eV for A excitons)	Large exciton binding energies, high oscillator strengths, valley-selective optical transitions, and van der Waals integration	Small active volume, limited single-pass absorption, exciton broadening, non-radiative recombination, and exciton–exciton annihilation	[31,44]

### 2.1. Band Structure and Optical Properties

**Figure 2:**
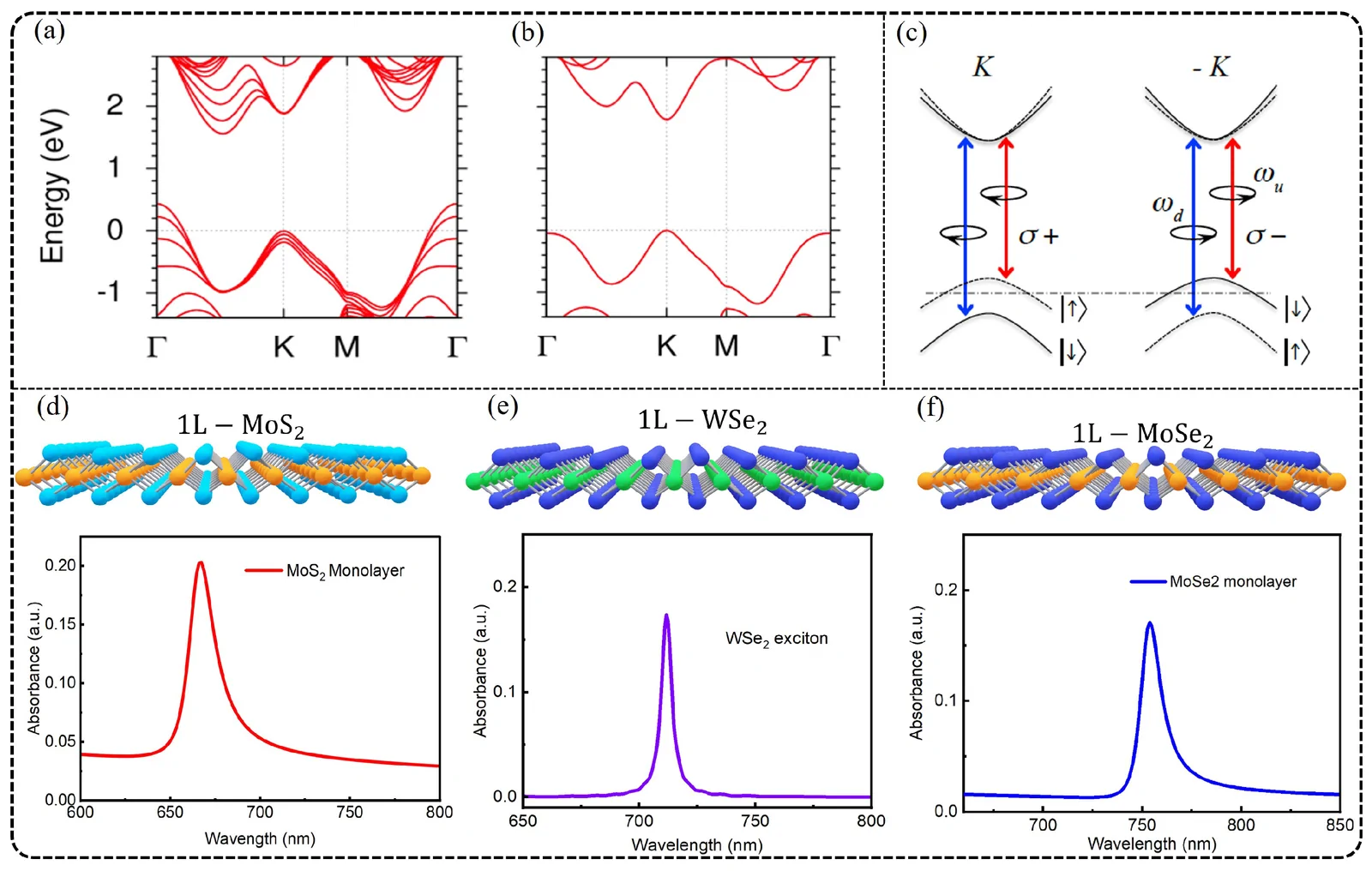
Evolution of the electronic and excitonic properties of atomically thin TMDCs. (**a**) Electronic band structure of multilayer ${\mathrm{WS}}_{2}$, illustrating the indirect nature of the fundamental band gap. (**b**) Electronic band structure of monolayer ${\mathrm{WS}}_{2}$, for which the fundamental gap becomes direct at the *K* valley as the material approaches the monolayer limit. Reproduced from [45], with permission from Springer Nature. (**c**) Schematic representation of spin–valley locking at the time-reversed *K* and $K^{\prime }$ (−K) valleys, showing the spin-split bands and the valley-dependent circularly polarised optical transitions $\sigma^{+}$ and $\sigma^{-}$. Reprinted figure with permission from [49], © 2012 American Physical Society. (**d**–**f**) Schematics and their corresponding modelled absorbance of monolayers: (**d**) ${\mathrm{MoS}}_{2}$, (**e**) ${\mathrm{WSe}}_{2}$, and (**f**) ${\mathrm{MoSe}}_{2}$. Reproduced with permission from ref. [50], © 2024, IEEE. Figure (**e**) reproduced from ref. [18] under CC BY 4.0.

The optical properties of TMDCs change substantially as their thickness decreases toward the monolayer limit. As illustrated in Figure 2a,b, reducing the number of layers modifies the relative energies of the electronic states across the Brillouin zone and drives the fundamental band gap from indirect in the multilayer material to direct in the monolayer [45]. In particular, the direct band edge transition of the monolayer occurs at the inequivalent *K* and $K^{\prime }$ (−K) valleys. This indirect-to-direct crossover is responsible for the pronounced enhancement in photoluminescence observed in monolayer TMDCs compared with their bulk or multilayer counterparts [46,47,48]. The monolayer limit also introduces important spin–valley physics. Broken inversion symmetry, together with strong spin–orbit coupling and time reversal symmetry, produces spin-split electronic states with opposite spin ordering at the time-reversed *K* and $K^{\prime }$ valleys [48,49]. As illustrated schematically in Figure 2c, the two valleys are associated with opposite circularly polarised optical selection rules, conventionally written as $\sigma^{+}$ and $\sigma^{-}$ transitions. The spin–orbit splitting of the valence band additionally gives rise to the characteristic A- and B-exciton transitions of monolayer TMDCs. Because of the reduced dielectric screening and strong quantum confinement in two dimensions, electron–hole interactions are particularly strong, resulting in robust excitonic resonances with large binding energies and oscillator strengths [31,44]. These properties make monolayer TMDCs particularly attractive as active excitonic media for strong light–matter coupling. For electromagnetic modelling, the dominant excitonic transition can be incorporated directly into the monolayer’s dielectric response. Figure 2d–f represent the A-exciton using a single Lorentz oscillator, for which the energy-dependent permittivity can be expressed as

(1) $$\varepsilon \left(E\right)=\varepsilon_{B}+\frac{f}{E_{\mathrm{exciton}}^{2}-E^{2}-i\Gamma E},$$

where *f* is the oscillator strength, $\varepsilon_{B}$ is the background dielectric constant, $E_{\mathrm{exciton}}$ is the exciton energy, and Γ is the width of the excitons. The resulting resonant and dispersive dielectric response provides a convenient material model for introducing the TMDC exciton into full-wave simulations and subsequently investigating its interaction with a resonant photonic or plasmonic mode.

### 2.2. The 2D Advantage and Its Limitation

The principal two-dimensional advantage of monolayer TMDCs is the combination of a strong excitonic response per unit thickness with the complete accessibility of the active layer. Unlike conventional quantum wells, which are typically embedded during epitaxial growth, a TMDC monolayer can be transferred directly to the electric field maximum of a prefabricated resonator. Its dangling-bond-free surface also permits integration with metals, dielectrics, and other van der Waals materials. The dielectric environment, carrier density, strain, and interlayer separation can therefore be engineered with considerable independence from the resonator geometry.

The same atomically thin geometry also imposes a fundamental limitation. Because the monolayer is less than one nanometre thick (≈0.7 nm), it overlaps with only a small fraction of an extended optical mode. Consequently, despite its large areal oscillator strength, the normal-incidence absorbance is generally only a few percent and may reach approximately 10–15% near a strong A-exciton resonance. For example, for an ideal reciprocal two-dimensional dipole sheet in a symmetric dielectric environment, the absorbance can be written as

(2) $$A=\frac{2\gamma_{r}\gamma_{\mathrm{nr}}}{{\left(\gamma_{r}+\gamma_{\mathrm{nr}}\right)}^{2}},$$

where $\gamma_{r}$ is the total radiative decay rate into the two half-spaces, and $\gamma_{\mathrm{nr}}$ is the nonradiative decay rate. The single-sided absorbance is bounded by 50% and reaches this limit only at critical coupling, $\gamma_{r}=\gamma_{\mathrm{nr}}$. Higher absorption therefore requires an asymmetric environment, a back-reflector, coherent excitation from both sides, or resonant field confinement that increases the effective interaction length [31,51]. These limitations can be mitigated first through chemical passivation, hBN encapsulation, electrostatic control, and the improved overall optical quality of the monolayer [52]. Resonant photonic structures instead enhance the radiative decay rate through the Purcell effect and can additionally improve directional out-coupling and the collection efficiency.

As a result, a large exciton-binding energy ensures that excitons remain stable at room temperature, but it does not guarantee a narrow linewidth, high efficiency, or a long coherence time. For instance, exciton–phonon scattering and inhomogeneous broadening increase the excitonic linewidth and reduce optical coherence. Heating can also reduce the effective oscillator strength and saturate the optical response. Subsequently, strong light–matter interaction in TMDC monolayers is therefore determined not by exciton stability alone but by whether the coherent interaction rate exceeds the relevant excitonic and photonic loss rates. These limitations motivate integrating TMDC monolayers with resonant metasurfaces. Here, a metasurface does not remove intrinsic material loss; rather, it increases the fraction of the optical field interacting with the monolayer by engineering the field distribution, effective mode volume, local density of optical states, and radiation channels (see Section 3 for how light is manipulated using metasurfaces).

## 3. Engineering Nanocavities for Tailored Light–Matter Interactions

Although monolayer TMDCs exhibit high excitonic oscillator strength, their small overlap between the optical mode and the atomically thin active layer fundamentally limits light–matter interaction. Engineering nanophotonic resonators seeks to overcome this limitation by maximising the overlap between the optical mode and the monolayer while simultaneously tailoring the mode volume, quality factor, radiative losses, and spectral detuning. This section introduces the fundamental design principles for engineering nanocavities that enhance light–matter interactions when coupled to TMDC monolayers in Section 4.

### 3.1. Design Principles for Subwavelength Light Confinement

The strength of light–matter interaction in a nanophotonic resonator depends on both the photon storage time and field confinement, commonly characterised by the quality factor *Q* and effective mode volume $V_{\mathrm{eff}}$. These quantities must be considered together: a long-lived mode offers a limited benefit if its field poorly overlaps the active material [53,54]. Because practical resonators exchange energy with their surroundings, their modes can be described by complex eigenfrequencies $\tilde{\omega }=\omega_{0}-i\Gamma /2$, where $\omega_{0}$ is the resonance frequency and Γ is the energy decay rate [55]. The quality factor of a Lorentzian resonance is then given by

(3) $$Q=2\pi \frac{\mathrm{energy}\;\mathrm{stored}}{\mathrm{energy}\;\mathrm{lost}\;\mathrm{per}\;\mathrm{cycle}}=\frac{\omega_{0}}{\Gamma }≃\frac{E_{0}}{\Delta E_{\mathrm{FWHM}}}.$$

For an asymmetric Fano feature, the peak or dip width need not equal the underlying modal linewidth because the resonant and non-resonant pathways interfere. An isolated Fano resonance may instead be fitted using

(4) $$T\left(\omega \right)=T_{\mathrm{bg}}+T_{0}\frac{{\left[q+2(\omega -\omega_{0})/\Gamma \right]}^{2}}{1+{\left[2(\omega -\omega_{0})/\Gamma \right]}^{2}},$$

where $T_{\mathrm{bg}}$ is the background transmittance, $T_{0}$ is the resonance amplitude, and *q* describes the asymmetry [3,56]. For a well-isolated resonance with a slowly varying background, the fitted Γ represents its modal linewidth and yields $Q=\omega_{0}/\Gamma$. The stored energy decays as exp(−Γt), giving an energy lifetime of

(5) $$\tau =\frac{1}{\Gamma }=\frac{Q}{\omega_{0}}.$$

Thus, one energy decay time corresponds to approximately Q/(2π) optical periods. If radiation and material dissipation are treated as independent loss channels,

(6) $$\Gamma =\Gamma_{\mathrm{rad}}+\Gamma_{\mathrm{dis}},\;\frac{1}{Q}=\frac{1}{Q_{\mathrm{rad}}}+\frac{1}{Q_{\mathrm{dis}}},$$

with $Q_{i}=\omega_{0}/\Gamma_{i}$ [4]. The geometry and symmetry primarily control radiative coupling (Section 3.2), whereas material absorption limits the dissipative contribution, particularly in plasmonic resonators (Section 3.3). Roughness, disorder, and finite-array effects introduce additional leakage or scattering, further reducing the measured *Q*.

Furthermore, in plasmonic nanocavities, metal absorption limits the achievable quality factor despite strong subwavelength field confinement. For a quasistatic localised surface plasmon resonance (LSPR), the material-limited quality factor can be estimated as $Q_{\mathrm{dis}}=\omega_{0}\left(d\varepsilon^{\prime }/d\omega \right)/\left(2\varepsilon^{\prime \prime }\right)$, where $\varepsilon^{\prime }$ and $\varepsilon^{\prime \prime }$ denote the real and imaginary parts of the metal permittivity [57]. The Drude relaxation times reported for Au and Ag are 14±3 fs and 17±3 fs, respectively, corresponding to calculated damping energies of approximately 0.047 and 0.039 eV [58,59]. As a result, individual visible-range LSPRs commonly exhibit Q∼5–10, whereas collective surface lattice resonances (SLRs) can reach substantially higher values through reduced radiative losses [60]. For example, experimentally reported SLR quality factors include Q=270 for colloidal nanoparticle arrays [61] and Q=2340 for Au nanoparticle arrays near λ=1.55μm [62]. The attainable *Q* depends on the operating wavelength and resonance geometry, particularly because Au exhibits substantial interband absorption below 500 nm (ℏω≳2.48 eV), whereas Ag generally shows lower interband absorption across the visible spectrum [63]. In comparison, all-dielectric metasurfaces supporting quasi-BICs have demonstrated higher quality factors in the visible and near-infrared spectral regions. These results highlight the trade-off between field confinement and optical losses in nanocavity design.

For a low-loss, weakly dispersive, well-confined mode, a conventional estimate of the effective mode volume is:

(7) $$V_{\mathrm{eff}}≃\frac{\int_{V}\varepsilon \left(r\right){\left|E\left(r\right)\right|}^{2}\;d^{3}r}{\max_{r}\left[{\varepsilon \left(r\right)|E\left(r\right)|}^{2}\right]}.$$

Here, ε(r) is the spatially dependent permittivity, E(r) is the local electric field at position r, and ${\left|E\left(r\right)\right|}^{2}$ is the electric field intensity. This is particularly relevant for atomically thin materials, for which strong light–matter interaction requires substantial spatial overlap between the resonant field and the monolayer.

### 3.2. Dielectric and High-Q Resonances

High-index dielectric nanostructures confine light through resonant displacement currents and, within low-absorption spectral regions, support electric and magnetic Mie-type resonances with substantially lower dissipative loss than plasmonic resonators [7]. Interference between their multipolar modes enables control of the far-field radiation and near-field distribution. Periodic arrangements introduce coupling between neighbouring resonators and in-plane Bloch modes, producing guided-mode resonances (GMRs) when a guided mode is coupled to the radiation continuum. Its interference with the non-resonant background generates the asymmetric Fano lineshape introduced in Section 3.1 [56]. The resonance wavelength and dispersion can be tuned through the lattice period, slab thickness, and meta-atom geometry.

Radiative loss can be further suppressed using optical bound states in the continuum (BICs). A BIC is a spatially confined eigenmode embedded in the radiation continuum but decoupled from the available far-field channels by symmetry or destructive interference [9]. In an ideal lossless and infinite structure, its radiative quality factor diverges, but the mode cannot be accessed directly through the corresponding free-space channel. Breaking the protecting symmetry converts it into a measurable quasi-BIC with a finite linewidth, allowing its radiative *Q* to be controlled geometrically.

In practice, the total *Q* is limited by absorption, surface roughness, structural disorder, substrate asymmetry, and the finite metasurface size, as described by Equation (6). Moreover, suppressing radiation too strongly reduces both the excitation and out-coupling efficiencies. Active devices therefore require an intermediate radiative coupling strength that balances photon storage, intrinsic loss, optical pumping, and emission extraction, rather than simply maximising *Q*.

This trade-off is particularly important for TMDC monolayers. Although a dielectric resonance may store substantial energy inside the high-index resonator, the atomically thin excitonic medium lies at its surface. The global cavity mode volume may therefore underestimate the effective mode volume experienced by the exciton. The relevant quantity is the accessible in-plane electric field at the monolayer, as defined by Equation (7). Gaps, slots, thin membranes, and appropriately shaped meta-atoms can shift the field maximum toward the surface and improve exciton–mode overlap. BIC and quasi-BIC resonances reduce the photonic linewidth, but strong coupling still requires sufficient field overlap and excitonic oscillator strength [10]. Dielectric metasurfaces therefore enhance $Q/V_{\mathrm{eff}}$ mainly by suppressing radiative loss while retaining low absorption. Their mode volumes are generally larger than those of plasmonic gaps, and their strongest fields may be less accessible to a surface-mounted monolayer. Consequently, a high cavity *Q* alone does not guarantee strong light–matter coupling.

### 3.3. Plasmonic Nanocavities and Resonances

Plasmonic confinement mechanisms can be organised by how a mode couples to the surrounding continuum. The LSPR of an isolated nanoparticle is highly radiative. Its dipolar current couples well to free space, and radiative damping, together with ohmic absorption, sets a limit [4,64]. The radiative channel must therefore be suppressed if any high-*Q* metallic structure is to be achieved. Radiative loss can be reduced by engineering weakly radiating multipoles, arranging meta-atoms so that their far-field scattering interferes destructively, or coupling adjacent metallic surfaces to confine the field within a dielectric gap [4,65]. The latter two approaches are the most relevant here.

The array-based approach produces surface lattice resonance (SLRs). When the local resonance of a periodic array of nanoparticles is spectrally matched to a diffracted order in the plane of the lattice, the particles scatter coherently, radiative damping is strongly suppressed, and the linewidth narrows substantially, enabling *Q* factors of $10^{2}$ and, in optimised structures, approaching $10^{3}$ [60,66,67]. The resulting mode is delocalised over many unit cells, and the effective mode volume is correspondingly large, which, for a single localised emitter, can substantially reduce the emitter–field coupling. For an extended two-dimensional active layer, this penalty is less severe. Writing the collective coupling as $g_{N}=g_{1}\sqrt{N}$, with $g_{1}\propto V^{-1/2}$ and $V≃AL_{\mathrm{eff}}$, an emitter sheet of areal density $n_{2D}$ contributes $N=n_{2D}A$; the mode area then cancels, giving $g_{N}\propto \sqrt{n_{2D}/L_{\mathrm{eff}}}$, so that the vertical confinement becomes the relevant geometrical factor. This is the reason that plasmonic lattices are able to support collective polariton condensation and lasing at accessible thresholds [68,69]. On the other hand, their spatially extended modes are generally less favourable for efficient coupling to a single localised quantum emitter. As a result, single-photon sources (SPSs) are not usually based on SLRs, since strong coupling to a single emitter demands a small mode volume and strong local field confinement. Other practical limitations include strong angular dispersion and, in many high-*Q* implementations, sensitivity to refractive index asymmetry between the substrate and superstrate.

The gap-based approach is realised in metal–insulator–metal (MIM) geometries. At a single interface, the SPP field decays evanescently into the dielectric as $\exp(-k_{d}x)$, with $k_{d}={\left(\beta^{2}-\varepsilon_{d}k_{0}^{2}\right)}^{1/2}$ being the transverse decay constant and β being the propagation constant. Bringing two interfaces within a decay length allows their evanescent fields to overlap, so that a symmetric transverse field profile can be written as

(8) $$\left|E\left(x\right)\right|\propto e^{-k_{d}x}+e^{+k_{d}x}=2\cosh\left(k_{d}x\right),$$

a catenary-like profile of the form acosh(x/b), in which $b=1/k_{d}$ is the scale parameter and *a* is the amplitude [70,71]. Narrowing the gap strengthens the coupling between the opposing surface modes. It increases the field confinement within the dielectric gap, producing a large local field enhancement while limiting the fraction of modal energy residing inside the metal [4,72]. In the catenary plasmonic metasurface reported by Berhe et al. [15], the single-catenary profile is described by $E\left(x\right)=a\cosh[\left(x\pm x_{0}\right)/b]+c$, where $x_{0}$ denotes the position of each catenary within the unit cell. The *Q* factor reaches approximately 35 for a narrower gap, compared with Q≈20.2 reported for isolated silver nanoprisms under ambient conditions [15,73]. This is in contrast to the normal compromise between confinement and loss. Over the range studied, the geometry reduces radiative leakage while concentrating the field in the dielectric gap, allowing both Q and $V_{\mathrm{eff}}^{-1}$ to increase simultaneously. However, this trend cannot continue indefinitely, as nonlocal effects introduce additional loss mechanisms at subnanometre gap sizes. Since the figure of merit for cavity-enhanced local light–matter interaction scales as $Q/V_{\mathrm{eff}}$, designs that simultaneously increase *Q* and reduce $V_{\mathrm{eff}}$ are particularly promising. The two families are therefore complementary rather than competing: SLRs provide extended, high-*Q* collective modes favourable for coherent polariton emission, whereas catenary gap cavities provide the strong local confinement required to couple efficiently to a single localised exciton.

Together, dielectric and plasmonic metasurfaces, including individual nanoantennas and periodically arranged resonant structures, provide distinct approaches for engineering and enhancing light–matter interactions at the nanoscale. Thus, they provide an insightful framework for selecting an appropriate nanocavity architecture and assessing whether a particular metasurface resonance offers sufficiently strong electromagnetic confinement and sufficiently low optical loss to reach the strong light–matter coupling regime. These considerations underpin Section 4, which examines strong coupling in TMDC–metasurface systems through the balance between coherent exciton–photon coupling and dissipative linewidths.

## 4. Strong Coupling in TMDC Monolayer–Nanocavities

### 4.1. Theoretical Foundation

Strong coupling occurs when coherent energy exchange between a photonic/plasmonic mode and an exciton outpaces their damping, producing hybrid light–matter states called polaritons [74]. This interaction can be described by a two-mode coupled-oscillator model, in which the bare photonic/plasmonic and excitonic resonances are linearly coupled with the strength, *g* [15,75]. Consider a photonic/plasmonic cavity mode described by the annihilation (creation) operators $\hat{a}$ (${\hat{a}}^{†}$) and a collective exciton mode described by $\hat{b}$ (${\hat{b}}^{†}$), where the excitonic mode is approximately bosonic in the low-excitation-density regime. Setting ℏ=1 and applying the rotating-wave approximation ($g\ll E_{P},E_{X}$), which neglects the counter-rotating terms $\hat{a}\hat{b}$ and ${\hat{a}}^{†}{\hat{b}}^{†}$, gives [76]

(9) $$\hat{H}=E_{P}\;{\hat{a}}^{†}\hat{a}+E_{X}\;{\hat{b}}^{†}\hat{b}+g\left({\hat{a}}^{†}\hat{b}+{\hat{b}}^{†}\hat{a}\right),\;g≃\mu_{X}\sqrt{\frac{NE_{P}}{2\varepsilon_{0}\varepsilon_{r}V}},$$

where $E_{P}$ (or $\omega_{P}$) and $E_{X}$ (or $\omega_{X}$) are the bare resonance energies. Including phenomenological damping to account for losses in the photonic/plasmonic and excitonic modes, the coherent amplitudes $\psi =\left(\begin{array}{c} \langle \hat{a}\rangle \\ \langle \hat{b}\rangle \end{array}\right)$ obey

(10) $$i\frac{d\psi }{dt}=H_{-}\psi ,\;H_{-}=\left(\begin{array}{cc} E_{P}-i\Gamma_{P} & g\\ g & E_{X}-i\Gamma_{X} \end{array}\right),$$

where $\Gamma_{P},\Gamma_{X}>0$ are the half-widths at half-maximum (HWHM) damping rates of the uncoupled photonic/plasmonic and excitonic modes, respectively.

The sign of the imaginary terms depends on the time convention. For $\psi \left(t\right)\propto e^{-iE^{\left(-\right)}t}$, Equation (10) becomes an eigenvalue problem for $H_{-}$, and decaying modes have ImE(−)<0. Taking the complex conjugate of $\psi^{*}\left(t\right)\propto e^{+iE^{\left(+\right)}t}$ and writing $E^{\left(+\right)}={\left[E^{\left(-\right)}\right]}^{*}$ gives the equivalent problem

(11) $$\underset{H_{+}=H_{-}^{*}}{\underset{︸}{\left(\begin{array}{cc} E_{P}+i\Gamma_{P} & g\\ g & E_{X}+i\Gamma_{X} \end{array}\right)}}\left(\begin{array}{c} \alpha \\ \beta \end{array}\right)=E_{\mathrm{UP},\mathrm{LP}}^{\left(+\right)}\left(\begin{array}{c} \alpha \\ \beta \end{array}\right),$$

where UP and LP denote the upper and lower polaritons, respectively. Here, decaying modes have ImE(+)>0, since e+iE(+)t=eiReE(+)te−ImE(+)t. Both conventions therefore describe the same damping.

The components α and β are the photonic and excitonic amplitudes, respectively. In the lossless limit, the Hopfield coefficients describe the photonic and excitonic fractions of the polariton modes, satisfying ${\left|\alpha \right|}^{2}+{\left|\beta \right|}^{2}=1$. For the lower polariton, these fractions are given by [77]

(12) $$|\beta_{\mathrm{LP}}{|}^{2}=\frac{1}{2}\left(1+\frac{\Delta }{\sqrt{\Delta^{2}+4g^{2}}}\right),\;{\left|\alpha_{\mathrm{LP}}\right|}^{2}=\frac{1}{2}\left(1-\frac{\Delta }{\sqrt{\Delta^{2}+4g^{2}}}\right),\Delta =E_{P}-E_{X}.$$

**Figure 3:**
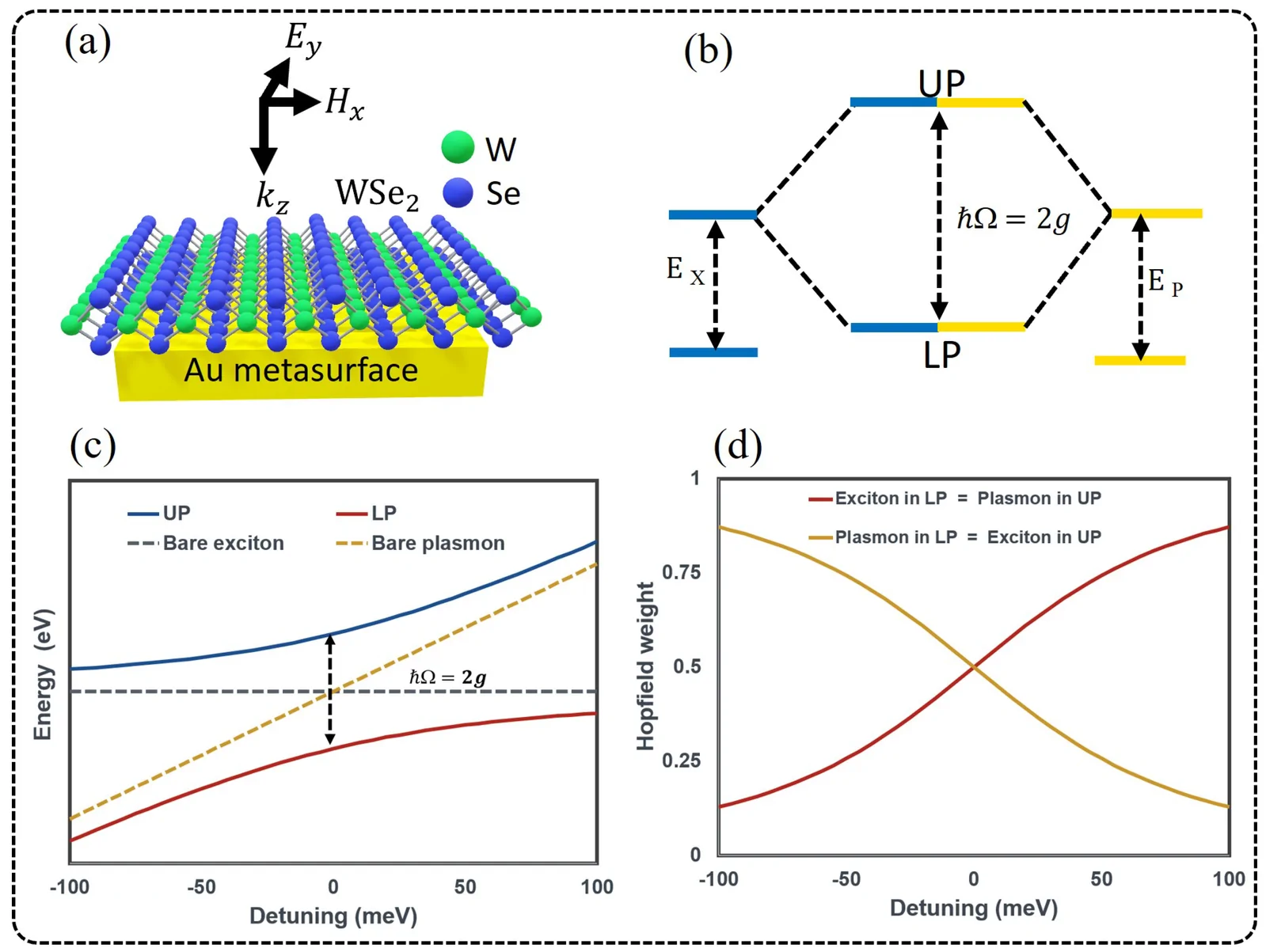
Formation of hybrid light–matter states. (**a**) Schematic of a ${\mathrm{MoSe}}_{2}$ monolayer placed atop an Au metasurface, under normally incident light polarised along the *y*-axis, and (**b**) the corresponding exciton and plasmon contribution s to the hybrid states. (**c**) Effect of varying the metasurface thickness on the strong coupling. (**d**) Hopfield coefficients showing the plasmon and exciton contributions to the hybrid states. Figure (**a**) reprinted from [15] licensed under CC BY 4.0.

As shown in Figure 3, the UP and LP form two energy branches that anticross as the detuning Δ is swept through zero [50]. With s=−1 for $H_{-}$ and s=+1 for $H_{+}$, the characteristic equation $\left(E_{P}+is\Gamma_{P}-E\right)\left(E_{X}+is\Gamma_{X}-E\right)-g^{2}=0$ gives

(13) $$E_{\mathrm{UP},\mathrm{LP}}^{\left(s\right)}=\frac{E_{P}+E_{X}+is\left(\Gamma_{P}+\Gamma_{X}\right)}{2}\pm \sqrt{g^{2}+\frac{1}{4}{\left[\Delta +is(\Gamma_{P}-\Gamma_{X})\right]}^{2}}.$$

Because $E_{P}$, $E_{X}$, *g*, and $\Gamma_{P,X}$ are real, the two spectra are complex conjugates, with identical real parts (modal energies) and opposite imaginary parts. At zero detuning, Equation (13) reduces to

(14) $$E_{\mathrm{UP},\mathrm{LP}}^{\left(s\right)}=E_{0}+\frac{is}{2}\left(\Gamma_{P}+\Gamma_{X}\right)\pm \sqrt{g^{2}-\frac{1}{4}{\left(\Gamma_{P}-\Gamma_{X}\right)}^{2}}.$$

When the square root is real, the Rabi splitting is

(15) $$\Omega =E_{\mathrm{UP}}^{\left(s\right)}-E_{\mathrm{LP}}^{\left(s\right)}=2\sqrt{g^{2}-\frac{1}{4}{\left(\Gamma_{P}-\Gamma_{X}\right)}^{2}}.$$

which is identical for both time conventions. At zero detuning, Ω represents the minimum separation between the real parts of the polariton eigenenergies, reducing to Ω=2g in the lossless limit. Nonzero Rabi splitting requires $g>|\Gamma_{P}-\Gamma_{X}|/2$. Furthermore, to resolve two distinct polariton resonances, the following two conditions should be satisfied [78]:

(16) $$g>\frac{|\Gamma_{P}-\Gamma_{X}|}{2},\;g>\sqrt{\frac{\Gamma_{P}^{2}+\Gamma_{X}^{2}}{2}}.$$

The second condition is more restrictive and ensures that the Rabi splitting exceeds the combined half-widths of the uncoupled resonances. Satisfying both conditions establishes spectrally resolved strong coupling within the coupled-oscillator model, characterised by distinct upper and lower polariton branches. Additionally, cooperativity is a way to verify the strong coupling regime. Hence, the key figure of merit is the light–matter coupling strength *g*, which, in the system, reaches tens of meV. This leads to high cooperativity $C=4g^{2}/\left(\kappa \gamma \right)>1$, placing the system firmly in the strong coupling regime [79,80]. Collective strong coupling establishes polariton formation but does not by itself demonstrate photon blockade, high-purity single-photon emission, or polariton lasing. These phenomena require additional nonlinear dynamics and independent photon correlation or coherence measurements.

### 4.2. Strong Coupling in Dielectric Metasurfaces

Dielectric metasurfaces have emerged as a particularly attractive design for achieving strong light–matter coupling with TMDC monolayers, combining negligible ohmic loss with subwavelength control of the electromagnetic near-field. As a result, several distinct resonance mechanisms have been exploited to reach the strong coupling regime in dielectric metasurface–TMDC systems. Guided-mode resonance (GMR) metasurfaces couple free-space radiation into the leaky guided modes of a periodically patterned dielectric slab, producing sharp Fano-type features whose strong dispersion affords both tunable photonic dispersion and directional polariton emission. As a result, Chen et al. experimentally demonstrated strong coupling between the A-exciton of monolayer ${\mathrm{WSe}}_{2}$ and GMRs supported by an all-dielectric SiN metasurface [81]. The metasurface consists of a two-dimensional square lattice of subwavelength holes fabricated in a SiN slab on a ${\mathrm{SiO}}_{2}$ substrate, and, on the surface of this slab, an hBN-encapsulated ${\mathrm{WSe}}_{2}$ monolayer is transferred directly so as to achieve evanescent coupling; the GMR not only confines part of the electromagnetic field within the dielectric slab but also preserves radiative coupling with the far-field. In the case of the uncoupled metasurface, a typical GMR at an energy of $E_{0}=1.726\;\mathrm{eV}$ had a quality factor of about Q∼143. This led to experimentally observed Rabi splitting of ℏΩ=18meV. The main strong coupling experiment was carried out at 22 K, with another sample exhibiting anticrossing at 80 K. Since ${\mathrm{WSe}}_{2}$ interacts only with the evanescent field at the SiN surface, a large portion of the GMR electromagnetic energy stored within the dielectric slab does not directly overlap with the atomically thin excitonic layer, and thus the achievable coupling is limited even though the dielectric resonance has low loss. To optimise the effect of field confinement and the position of TMDC monolayers when integrated with dielectric metasurfaces, we designed and theoretically investigated strong exciton–photon coupling between TMDC monolayers and an ultrathin lithium niobate (LN) metasurface supporting a tunable Fano-shaped photonic resonance [3]. The metasurface consists of narrow air gaps, which strongly confine the electromagnetic field within the dielectric structure and around the gap region. The *Q* factor is strongly influenced by the metasurface geometry, such as the gap and thickness. Hence, it decreases from about 140 to 110 as the LN thickness increases from 80 to 140 nm, due to higher radiative losses. As a result, thinner structures lead to better field confinement and higher *Q* factors. Accordingly, we investigated strong coupling for monolayer TMDCs using this field overlap strategy. Subsequently, a maximum ℏΩ=52.76meV in LN–${\mathrm{MoS}}_{2}$ was achieved when the ${\mathrm{MoS}}_{2}$ monolayer was placed at the electric field maximum of a high-*Q* dielectric metasurface at room temperature. However, although the demonstrated Rabi splitting is larger compared to other hybrid structures [82], it is smaller because the coupling strength $g\propto \sqrt{N/V}$.

Following the investigation of GMR-based strong coupling, we consider high-*Q* dielectric metasurfaces, particularly those supporting BIC and quasi-BIC platforms. For example, Kravtsov et al. experimentally coupled monolayer ${\mathrm{MoSe}}_{2}$ to a symmetry-protected optical BIC supported by a one-dimensional ${\mathrm{Ta}}_{2}$$O_{5}$ photonic crystal slab by using a 9 nm hBN spacer to spectrally tune the BIC toward the ${\mathrm{MoSe}}_{2}$ exciton energy of 1.65 eV, producing Rabi splitting of 27.4 meV at 7 K [83]. Even though the splitting was only comparatively moderate, the suppression of radiative leakage near the BIC allowed the polariton linewidths to fall below 3 meV and the Q factors to reach values approaching 900, thus showing the main benefit of BIC cavities—that is, long photon lifetimes rather than extremely small-scale field confinement.

**Figure 4:**
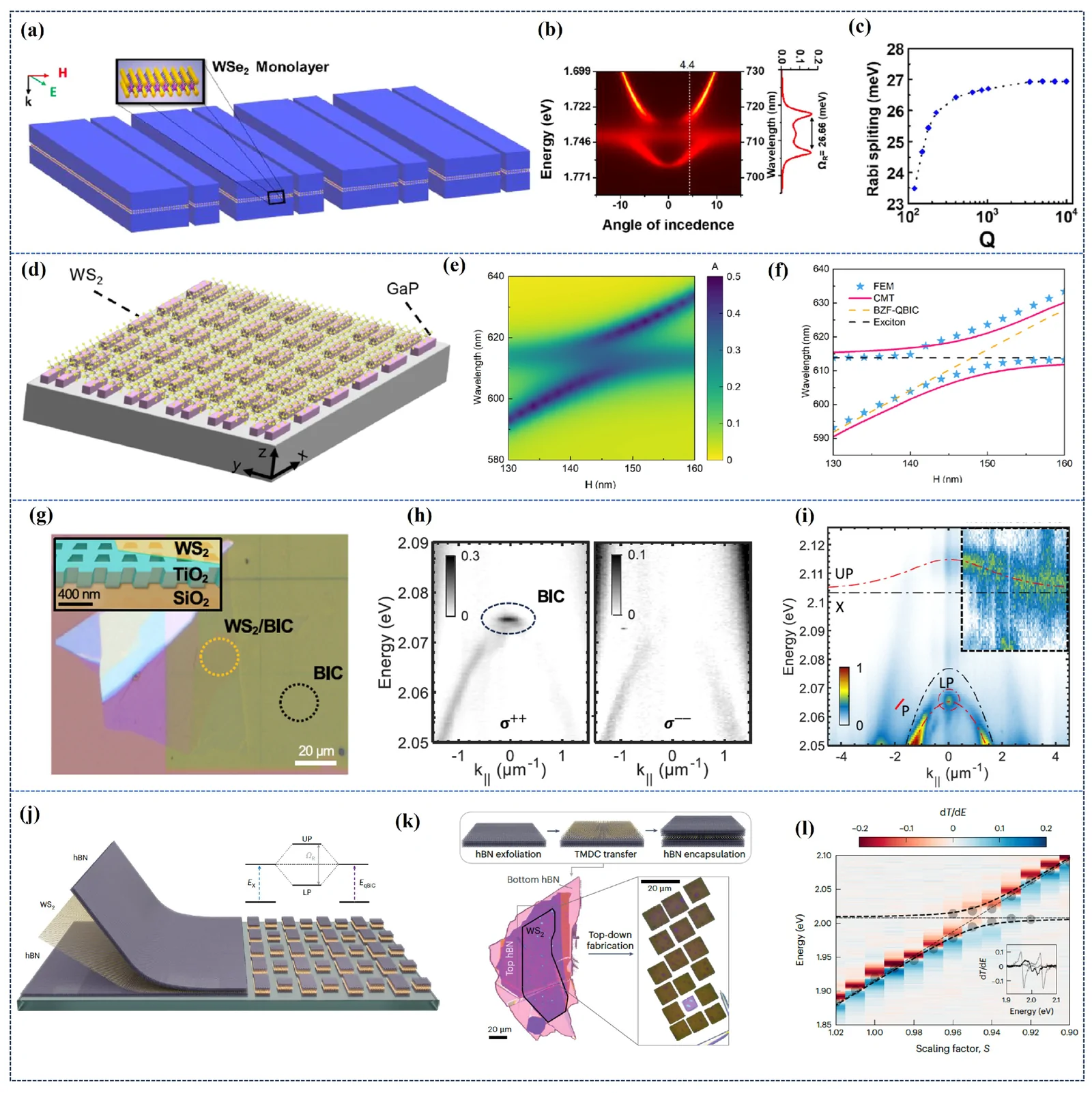
Strong coupling in dielectric metasurface–1L-TMDCs (**a**) Schematic of the hybrid structure, in which the ${\mathrm{WSe}}_{2}$ monolayer is embedded within ${\mathrm{Si}}_{3}$$N_{4}$ nanowires near the maximum of the qBIC electric field. (**b**) Angle-dependent absorption mapping showing pronounced anticrossing between the dispersive qBIC mode and the ${\mathrm{WSe}}_{2}$ exciton, resulting in upper- and lower-polariton branches with Rabi splitting of approximately 26.66 meV, and (**c**) the Rabi splitting as a function of the Q factor. Reproduced with permission from ref. [10], © 2021 Wiley-VCH GmbH. (**d**) Schematic of hybrid ${\mathrm{WS}}_{2}$–GaP metasurface consisting of monolayer ${\mathrm{WS}}_{2}$ positioned on periodic GaP nanorod/${\mathrm{SiO}}_{2}$ substrate. The structural perturbation folds the relevant guided mode into the radiation continuum, producing the BZF-qBIC. (**e**) Calculated absorption mapping as a function of the GaP nanorod height *H*, showing pronounced avoided crossing with clear UP and LP branches. (**f**) The corresponding extracted polariton energies show maximum Rabi splitting of approximately 38.2meV. Figure (**d**–**f**) reprinted from ref. [85], Copyright © 2026 American Physical Society. (**g**) Schematic of the hybrid structure, consisting of monolayer ${\mathrm{WS}}_{2}$ transferred onto a ${\mathrm{TiO}}_{2}$ metasurface designed to support a chiral BIC. (**h**) Polarisation-resolved reflectivity of the metasurface under opposite circularly polarised excitation, $\sigma^{+}$ and $\sigma^{-}$. The sharp resonance observed for $\sigma^{+}$ and strongly suppressed for $\sigma^{-}$ demonstrates the helicity-selective character of the chiral BIC, which exhibits a quality factor of approximately Q∼1500. (**i**) Normalised angle-resolved photoluminescence showing the UP and LP branches formed by strong coupling between the ${\mathrm{WS}}_{2}$ exciton and the chiral BIC, resulting in Rabi splitting of 2ℏΩ=42.0meV. Reproduced from ref. [86] under CC BY 4.0. (**j**) Schematic illustration of the hBN/${\mathrm{WS}}_{2}$/hBN van der Waals heterostructure, patterned as a metasurface that supports a qBIC and couples to the ${\mathrm{WS}}_{2}$ exciton. (**k**) Detailed experimental procedures of the qBIC unit-cell geometry, including the embedded position of ${\mathrm{WS}}_{2}$, 125 nm total thickness, and structural asymmetry. (**l**) By geometrically adjusting the qBIC with respect to the ${\mathrm{WS}}_{2}$ exciton, experiments measured the transmission spectra, showing an avoided crossing between the upper and lower polariton branches and Rabi splitting of 30 ± 1 meV at room temperature. Reproduced from ref. [87] under CC BY 4.0.

Additionally, as shown in Figure 4a,b, Al-Ani et al. demonstrated Rabi splitting of approximately 26.66 meV by varying the incident angle. By optimising the nanostructure thickness and *Q* factor, Rabi splittings of up to 38, 65, 40, and 60 meV were obtained for ${\mathrm{WSe}}_{2}$, ${\mathrm{WS}}_{2}$, ${\mathrm{MoSe}}_{2}$, and ${\mathrm{MoS}}_{2}$ monolayers, respectively, coupled to magnetic dipole and toroidal dipole BIC modes [10]. A related ${\mathrm{Si}}_{3}$$N_{4}$–${\mathrm{WS}}_{2}$ quasi-BIC metasurface demonstrated room-temperature strong coupling with Rabi splitting of up to 28.4 meV when the ${\mathrm{WS}}_{2}$ monolayer was positioned at the maximum local field plane, further demonstrating that both the resonance linewidth and local electric field enhancement must be balanced when optimising exciton–photon coupling [84]. These studies therefore demonstrate that an ultrahigh *Q* factor does not automatically produce proportionally large Rabi splitting. Indeed, in the BIC-TMDC study [10], the Rabi splitting increases with *Q* only until the local field enhancement saturates, showing that further suppression of radiative loss does not indefinitely increase the coupling strength in the system.

Recently, Guo et al. proposed a dielectric metasurface–${\mathrm{WS}}_{2}$ monolayer system supporting strong coupling through a Brillouin-zone folding-induced quasi-BIC (BZF-qBICs) [85]. As shown in Figure 4d, the ${\mathrm{WS}}_{2}$ monolayer is placed on a GaP nanorod metasurface supported by ${\mathrm{SiO}}_{2}$. Perturbing the spacing between adjacent nanorods doubles the periodicity along one direction, folding a guided mode from the Brillouin-zone edge to the Γ point and enabling its coupling to free-space radiation. Unlike conventional asymmetry-induced qBICs, the BZF-qBIC allows control of the linewidth and *Q* factor with minimal resonance wavelength shift, preserving spectral matching with the exciton. By varying the nanorod height *H*, the BZF-qBIC resonance was tuned across the ${\mathrm{WS}}_{2}$ A-exciton, producing an avoided crossing and upper and lower polariton branches, as shown in Figure 4e,f. The simulations yielded maximum Rabi splitting of 38.2 meV and coupling strength g=19.5 meV, with excitonic and cavity dissipation energies of 14 and 6.1 meV, respectively, satisfying the strong coupling criteria. The electric quadrupole-dominated BZF-qBIC also provides localised field enhancement, with the strongest coupling obtained when ${\mathrm{WS}}_{2}$ is positioned on top of the nanorods.

Later, Wurdack et al. also experimentally demonstrated strong coupling between valley excitons in monolayer ${\mathrm{WS}}_{2}$ and a chiral BIC supported by an all-dielectric ${\mathrm{TiO}}_{2}$ metasurface [86]. As illustrated in Figure 4g, the hybrid structure consists of an exfoliated ${\mathrm{WS}}_{2}$ monolayer transferred onto a 220 nm thick ${\mathrm{TiO}}_{2}$ layer patterned with a square array of slant-perturbed trapezoidal nanoholes on a ${\mathrm{SiO}}_{2}$ substrate. The symmetry perturbation of the metasurface generates a highly polarisation-selective chiral BIC, whose chiral character is evident from the polarisation-resolved angle-dependent reflectivity shown in Figure 4h. At a normal incidence of $k_{\|}=0$, a narrow resonance is induced at 2.075eV for $\sigma^{+}$-polarised excitation, whereas the corresponding resonance disappears for $\sigma^{-}$-polarised excitation. Here, $\sigma^{+}$ and $\sigma^{-}$ denote the two opposite circular polarisation helicities. This strong helicity selectivity is a signature of the chiral BIC, which exhibits a quality factor of approximately Q∼1500. The polarisation selectivity is particularly relevant for monolayer ${\mathrm{WS}}_{2}$, since opposite circular polarisations selectively address the *K* and $K^{\prime }$ valley excitons. Consequently, the metasurface provides not only a high-*Q* optical mode to enhance exciton–photon interactions but also a means to control the spin and valley character of the resulting hybrid states. As clearly shown in Figure 4i, this hybrid structure exhibits strong coupling, with Rabi splitting of ℏΩ=42.0meV. It should be noted that the strong coupling measurements were performed at cryogenic temperatures (T≈4K). The Rabi splitting at cryogenic temperatures could be due to the field distribution inside the ${\mathrm{TiO}}_{2}$ metasurface, while the ${\mathrm{WS}}_{2}$ monolayer is on the top [3,10].

More recently, Sortino et al. experimentally demonstrated room-temperature strong coupling in an ultrathin van der Waals heterostructure metasurface formed from a monolayer ${\mathrm{WS}}_{2}$ encapsulated between hBN layers [87]. As demonstrated in Figure 4j,k, in contrast to conventional dielectric metasurface–TMDC hybrids, where the monolayer is transferred onto the surface of a prefabricated resonator, the hBN/${\mathrm{WS}}_{2}$/hBN heterostructure itself was nanopatterned into an approximately 125 nm thick dielectric metasurface composed of asymmetric nanorod pairs. Because of its asymmetric design, a radiative channel is formed for a symmetry-protected qBIC, leading to Fano-like high-*Q* resonances with strong in-plane electric fields that directly overlap with the ${\mathrm{WS}}_{2}$ excitons. In experiments, metasurfaces operating with detuned settings exhibited quality factors Q>100. Upon tuning the qBIC through the ${\mathrm{WS}}_{2}$ exciton, distinct anticrossing was observed both in the transmission and in the polaritonic photoluminescence, giving rise to Rabi splitting of ℏΩ=30±1meV at room temperature (see Figure 4l). This architecture therefore has the main advantage of achieving better spatial overlap between the dielectric cavity field and the atomically thin excitonic layer without losing the low loss properties of the dielectric qBIC resonance. Nevertheless, the cavity Q factor and the Rabi splitting observed in these experiments are only moderate. To obtain more coherent nonlinear effects, such as polariton lasing, the cavity Q must increase, and thermal dephasing must decrease.

Overall, the dielectric systems discussed above demonstrate that low-loss resonances can support strong exciton–photon coupling when the field overlaps sufficiently with the monolayer. The reported Rabi splittings are specific to the TMDC material, temperature, exciton linewidth, and device geometry used in each study, and they should not be treated as a general upper limit for dielectric metasurfaces. Plasmonic structures offer a complementary way to increase the field accessible to the monolayer, but their absorptive losses must also be considered.

### 4.3. Strong Coupling in Metallic Metasurfaces

The all-dielectric metasurfaces hybridised with TMDC monolayers discussed in Section 4.2 reach the strong coupling regime by maximising the photonic quality factor Q, confining light for many optical cycles while dissipating almost none of it. Plasmonic metasurfaces invert this strategy entirely. Their quality factors are one to two orders of magnitude lower, yet these metal nanoparticles and plasmonic arrays use plasmonic modes to confine strong electric fields in a small mode volume, enhancing interaction with TMDC monolayers and enabling Rabi splitting greater than 100 meV [65].

When we consider using metallic nanocavities for strong coupling investigations with TMDC monolayers, we need to consider different plasmonic resonances, and the choice among them sets the entire trade space. As a result, before proceeding to metasurface-based systems, we first highlight the strong coupling in localised SPRs of individual nanodisks, rods, or prisms, as well as gap plasmons in nanoparticle-on-mirror (NPoM), metal–insulator–metal, bowtie, and nanotrench geometries, which push V to its practical minimum and induce large Rabi splitting [76]. In this context, from the nanoantenna perspective, Han et al. demonstrated room-temperature strong plasmon–exciton coupling between a monolayer of ${\mathrm{WS}}_{2}$ and an ultracompact NPoM plasmonic nanocavity, consisting of a silver nanocube positioned above a silver film with an approximately 7 nm ${\mathrm{Al}}_{2}$$O_{3}$ spacer [88]. The surface plasmon resonance was tuned to match the ${\mathrm{WS}}_{2}$ excitons by changing the nanocube size and the nanograph thickness, resulting in two hybrid plexcitonic branches and clear anticrossing that confirmed that the system was in the strong coupling regime. The measured Rabi splitting was about 145 meV. Here, the strong coupling criterion was confirmed by substituting $\gamma_{\mathrm{SP}}=220\;\mathrm{meV}$ and $\gamma_{0}=50\;\mathrm{meV}$ to $\hbar \Omega >(\gamma_{\mathrm{SP}}+\gamma_{0})/2$. In addition to silver’s instability compared to gold, this broad ∼220meV plasmon linewidth results in a low *Q* factor.

**Figure 5:**
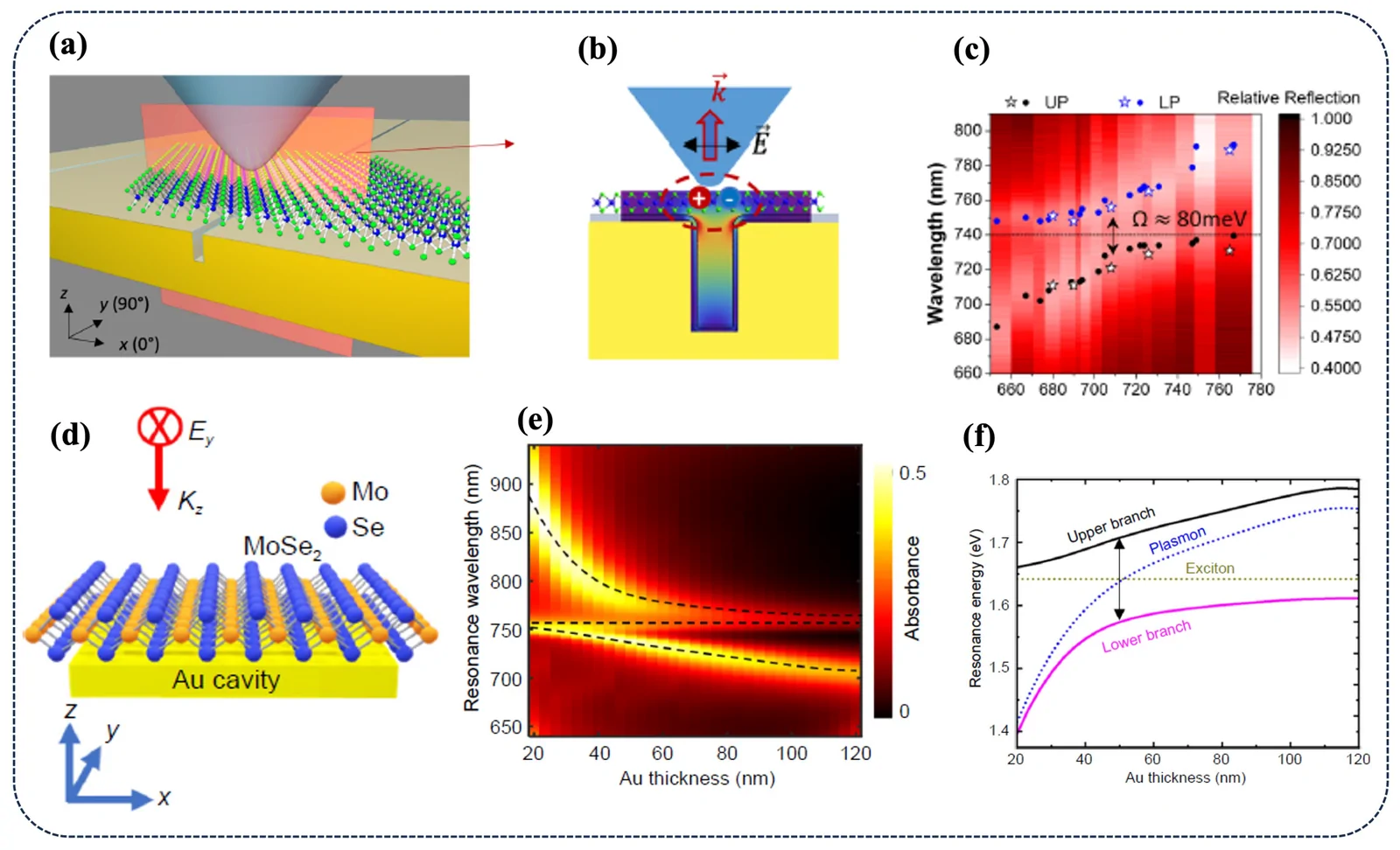
Catenary-shaped near-field enhancement in a plasmonic metamaterial cavity. (**a**) Unit cell of the Au metamaterial cavity, with design parameters $W_{1}=39$ nm, $W_{2}=33$ nm, h=157 nm, g=108 nm, α=307 nm, and t=60 nm. (**b**) Electric field distribution $|{E/E}_{0}|$ of the uncoupled cavity in the *z*-plane, showing the energy confined within the dielectric gap and concentrated at the air–gold boundaries. (**c**) Retrieved near-field enhancement $|{E/E}_{0}|$ across the 39 nm cavity gap, exhibiting the symmetric hyperbolic-cosine envelope characteristic of two evanescently coupled metal–dielectric interfaces, with maxima of 25.1 at the air–gold boundaries. Reproduced from ref. [89] under CC BY 4.0. (**d**) Quality factor of the cavity as a function of the gap width for lattice periods of 420 and 550 nm. Narrowing the gap raises *Q* to approximately 35, substantially above that of an isolated plasmonic nanoparticle, so the field confinement and photon lifetime improve together. Reproduced from ref. [15] under CC BY 4.0.

Another study by Zhou et al. investigated strong plasmon–exciton coupling between monolayer ${\mathrm{WSe}}_{2}$ and one-dimensional gold nanotrenches coated with a thin 2 nm ${\mathrm{Al}}_{2}$$O_{3}$ layer [89]. The nanotrenches support a strongly confined plasmon mode in the gap, analogous to the lowest-order SPP mode of an MIM waveguide. The designed nanocavity has a large bare plasmon linewidth of κ≈130 meV, which, at the ${\mathrm{WSe}}_{2}$ A-exciton energy ($E_{0}\approx 1.67$ eV), corresponds to a low quality factor of $Q\approx E_{0}/{\Delta E}_{FWHM}∼13$ (see Equation (3)). Here, the plasmon resonance was tunable by changing the trench width and depth to match the ${\mathrm{WSe}}_{2}$ exciton, resulting in Rabi splitting of ∼80 meV, close to the calculated value of 86.6 meV (see Figure 5a–c). This coupling is enabled by the strong in-plane electric field of the nanotrench design, which aligns with the in-plane excitonic dipole of ${\mathrm{WSe}}_{2}$ and facilitates efficient coupling. More importantly, despite the low Q factor, near-field PL measurements showed highly localised lower-plexciton emission, confined laterally to about 20 nm. Therefore, reducing plasmonic losses and further optimising the nanotrench geometry could improve control over nanoscale plexciton emission and enable dynamically tunable plexcitonic devices.

Another study by Wei et al. also demonstrated room-temperature strong plasmon–exciton coupling between monolayer ${\mathrm{WSe}}_{2}$ and lithographically defined single Au nanobowties. The bowtie supports localised surface plasmon (LSP) modes, with the longitudinal mode producing intense field confinement within an approximately 10 nm gap and therefore a very small effective mode volume. The resulting plasmon linewidth is κ≈204 meV, resulting in a Q factor of Q≈8.2. As a result, by varying the bowtie size, the plasmon resonance was tuned across the ${\mathrm{WSe}}_{2}$ A-exciton with a linewidth of 55 meV, producing clear anticrossing with the strong coupling of 187 meV and a coupling strength of 93.5 meV [90]. However, the large plasmon linewidth and the resulting low Q factor are still major limitations, and the isolated nanoparticle structure does not exhibit the collective optical response typical of a periodic plasmonic metasurface. On the other hand, its lithographic compatibility, together with strong local coupling, offers a useful pathway to developing compact, on-chip plexcitonic nanodevices.

Here, the radiative loss of plasmonic nanoantennas can be reduced by employing a subradiant bonding mode. In this regard, Wu et al. numerically demonstrated strong plasmon–exciton coupling between monolayer ${\mathrm{WS}}_{2}$ and a low-loss silver nanocuboid dimer (NCD) [91]. In contrast to conventional bright LSPR modes, the dimer exhibits a subradiant bonding plasmonic mode, which suppresses radiative leakage while still maintaining strong nanoscale field confinement, leading to a plasmon linewidth of about 60 meV, as compared to 168 meV for a single nanocuboid and 280 meV for the antibonding mode. However, this work showed that the Rabi splitting in the hybrid system was approximately 60 meV. The relatively modest Rabi splitting in the Ag NCD–${\mathrm{WS}}_{2}$ monolayer could be enhanced by further optimising the exciton–plasmon mode overlap and local field confinement. To go beyond simply demonstrating strong coupling, it will be essential to increase the Q factor while maintaining a small plasmonic mode volume to reduce optical losses and enhance the coherence and efficiency of the hybrid light–matter states.

As a result, periodic design is crucial for enhancing the Q factor. In this regard, Berhe et al. demonstrated that Au–${\mathrm{MoSe}}_{2}$ and Au–${\mathrm{WSe}}_{2}$ metamaterial cavities (metasurfaces) can sustain catenary-shaped plasmonic near-fields whose strength is controlled by the cavity gap and metallic thickness. The resulting deep-subwavelength localisation substantially improves the field sampled by the TMDC monolayer and produces calculated room-temperature Rabi splittings of >100 meV [15]. However, this comparison does not imply that plasmonic resonances are intrinsically superior to dielectric metasurfaces with BICs. Both systems achieve the strong coupling regime through fundamentally different light confinement mechanisms. The main question is which one could provide larger Rabi splitting in TMDC monolayers at room temperature. As explained above, metallic absorption imposes an intrinsic loss channel that limits the achievable Q and photon lifetime in plasmonic cavities. Rather, this comparison thus reveals the complementary roles of deep-subwavelength spatial confinement in plasmonic cavities and strong temporal confinement in dielectric BIC resonators. Hence, dielectric BICs predominantly enhance temporal confinement by suppressing radiative leakage. In contrast, plasmonic nanocavities can generate much stronger nanoscale spatial confinement and therefore a substantially larger electric field at the TMDC plane. The difference is therefore more consistently attributed to the combined effects of a reduced effective mode volume and enhanced local field overlap, rather than to the *Q* factor alone. Accordingly, a metasurface should be selected to achieve strong coupling with large Rabi splitting, which should be evaluated through the joint optimisation of the *Q* factor, $V_{\mathrm{eff}}$, $Q\mathrm{factor}/V_{\mathrm{eff}}$, the local enhancement $|E/E_{0}|$, spectral detuning, and the field overlap with the TMDC monolayer.

In this regard, SLRs in plasmonic metasurfaces can provide a favourable trade-off between a relatively high *Q* factor and subwavelength mode confinement, thereby improving the Q/V ratio while maintaining strong exciton–plasmon coupling.

As explained in Section 3.3, the Q factor of a plasmonic metasurface can be enhanced by designing it to exhibit SLR. Hence, these plasmonic BIC metasurfaces provide a route to combine the small effective mode volume of plasmonic resonances with the suppression of radiative leakage associated with a bound state in the continuum. In an ideal periodic structure, symmetry protection prevents the BIC from coupling to far-field radiation at the Γ point. In a metallic metasurface, however, the suppression of radiative loss does not eliminate ohmic absorption, which continues to limit the total quality factor. Consequently, the finite-linewidth mode accessed experimentally is more accurately regarded as a plasmonic quasi-BIC, although it is commonly referred to as a plasmonic quasi-BIC.

**Figure 6:**
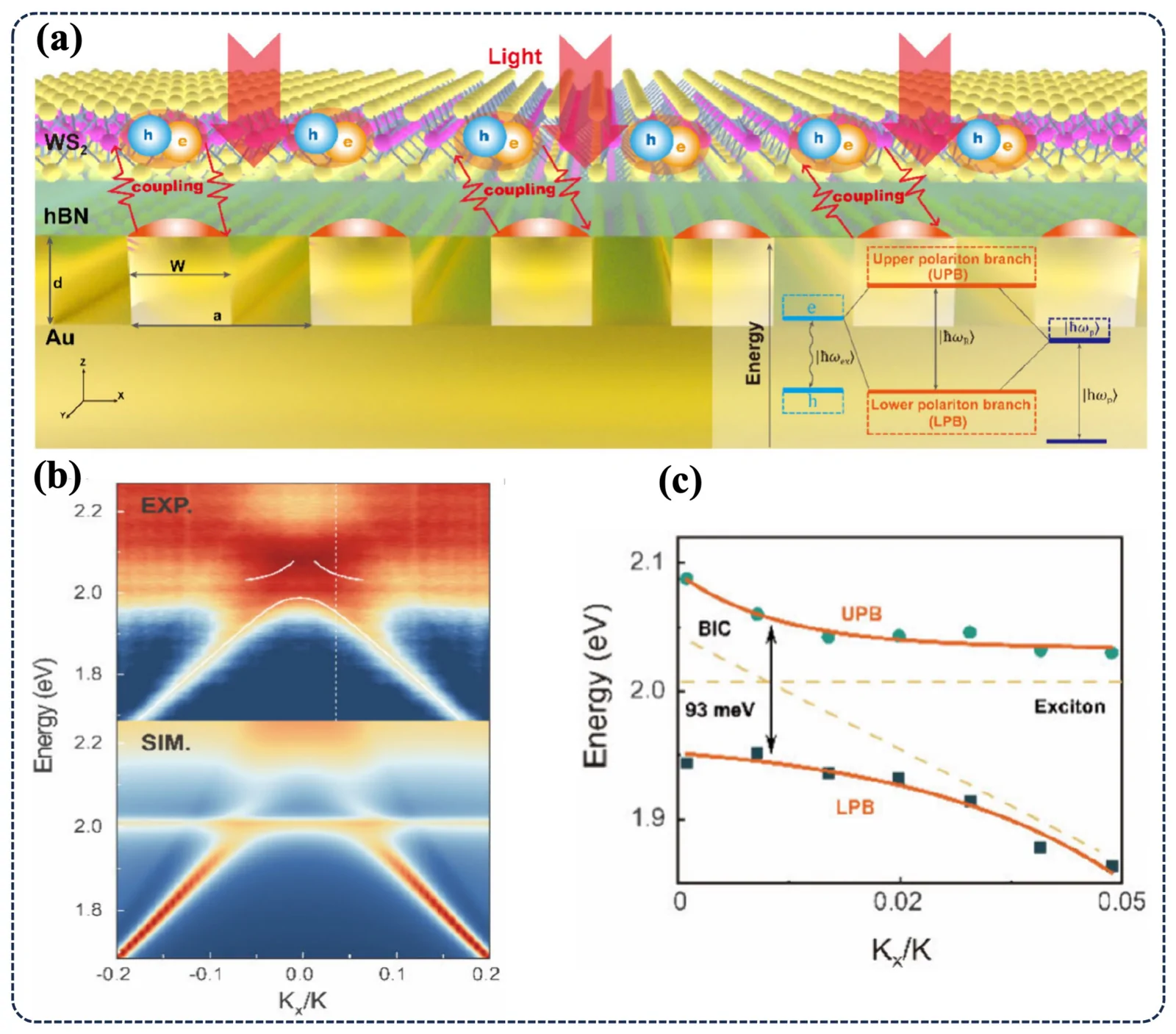
Room-temperature strong coupling between monolayer ${\mathrm{WS}}_{2}$ and a plasmonic quasi-BIC metasurface. (**a**) Schematic of the hybrid structure, consisting of monolayer ${\mathrm{WS}}_{2}$ integrated with an hBN-spaced one-dimensional Au grating supporting a plasmonic quasi-BIC. (**b**) Experimental angle-resolved reflectance spectrum showing anticrossing between the dispersive plasmonic mode and the ${\mathrm{WS}}_{2}$ A-exciton. (**c**) Extracted polariton dispersion fitted using the coupled-oscillator model, yielding Rabi splitting of approximately 93 meV at room temperature. Reproduced with permission from ref. [14]. Copyright © 2025 American Chemical Society.

Luo et al. reported room-temperature strong coupling between the A-exciton of monolayer ${\mathrm{WS}}_{2}$ and a plasmonic quasi-BIC supported by a one-dimensional Au grating [14]. As shown in Figure 6a, the ${\mathrm{WS}}_{2}$ monolayer was integrated with the Au grating through an hBN spacer. The calculated quality factor approached Q∼500 near the Γ point in the low-loss metal model. In the experiment, metal absorption reduced the resonance quality factor to approximately Q∼56. Angle-resolved reflectance measurements revealed clear anticrossing between the dispersive plasmonic mode and the ${\mathrm{WS}}_{2}$ A-exciton, as shown in Figure 6b. The coupled-oscillator fit in Figure 6c yielded Rabi splitting of approximately 93 meV, supporting the formation of upper- and lower-polariton branches at room temperature. The coupling in this system arises from the combined effects of reduced radiative leakage and plasmonic field confinement, rather than from a high *Q* factor alone. These measurements establish the linear strong coupling regime, while nonlinear polariton emission and polariton lasing were not investigated.

This example shows that plasmonic metasurfaces should not be assessed using either the *Q* factor or Rabi splitting alone. Their suitability for light emission applications depends on the joint optimisation of *Q*, $V_{\mathrm{eff}}$, $Q/V_{\mathrm{eff}}$, local field enhancement, spectral detuning, and the spatial and polarisation overlap between the resonant field and the TMDC exciton. These considerations provide a physical basis for selecting resonant platforms for the single-photon emission and lasing applications discussed in Section 5.

Table 2 shows comparative analysis of representative dielectric and plasmonic metasurface–TMDC monolayer systems exhibiting strong light–matter coupling and their implications for light emission applications.

## 5. Metasurface–TMDC Monolayers for Compact Light Sources

The light emission mechanisms discussed below belong to distinct physical regimes. In weak coupling, Purcell enhancement modifies the spontaneous emission rate of a localised emitter, while its single-photon character originates from the emitter itself. Collective strong coupling instead produces exciton–polariton modes from an extended excitonic ensemble and does not by itself establish single-photon emission. Single-emitter nonlinear strong coupling requires coupling to an individual quantum emitter and a resolvable anharmonic excitation spectrum for conventional photon blockade. Photon lasing relies on optical gain and stimulated emission into a cavity mode, whereas polariton lasing involves the bosonic stimulation of polariton-state occupation while maintaining strong exciton–photon coupling across the lasing threshold.

### 5.1. Single-Photon Sources (SPSs)

As a result, the temporal evolution of the photon statistics can be used to characterise photon purity using the normalised time-delayed second-order correlation function $g^{\left(2\right)}\left(\tau \right)$, where τ represents the delay between two photon detection events [18,93]:

(17) $$g^{\left(2\right)}\left(\tau \right)=\frac{\langle a^{†}\left(t\right)a^{†}\left(t+\tau \right)a\left(t+\tau \right)a\left(t\right)\rangle }{{\langle a^{†}a\rangle }^{2}},$$

where *a* ($a^{†}$) denotes the annihilation (creation) operator of the cavity mode. At sufficiently long delays, $g^{\left(2\right)}\left(\tau \right)\to 1$ as correlations between successive photon emission events decay and the detections become statistically uncorrelated. Experimentally, $g^{\left(2\right)}\left(\tau \right)$ is commonly measured using a Hanbury Brown–Twiss (HBT) interferometer, which records the temporal correlations between photon detection events at two detectors. These photons can be emitted by semiconductor quantum emitters, and demonstrating high-purity emission under ambient conditions is desired for quantum applications. Additionally, the brightness of these emitted photons can be described as the rate at which usable single photons are generated and extracted from the source and therefore depends on both the photon generation rate and the extraction efficiency. For far-field collection, the latter is set by the radiative quantum yield and the fraction of emitted photons collected within the optical system’s numerical aperture [93]. Indistinguishability also describes how identical consecutively emitted photons are in their spatial and temporal wave packets, spectra, and polarisation and is commonly quantified using Hong–Ou–Mandel (HOM) interference. Ideally, indistinguishable photons that arrive at a 50:50 beam splitter at the same time end up in the same output channel, resulting in a vanishing coincidence probability and HOM visibility approaching unity [94]. This review focuses more on photon purity because it directly shows whether the source produces single photons, as measured by $g^{\left(2\right)}\left(0\right)$, and helps to quantify how well unwanted multiphoton emission is suppressed.

In this context, in 2015, He et al. reported one of the pioneering demonstrations of single-photon emission from spatially localised excitons in monolayer ${\mathrm{WSe}}_{2}$ [16]. The experiment was conducted at 4.2 K without the integration of an optical nanocavity. Pronounced photon antibunching was measured, with $g^{\left(2\right)}\left(0\right)=0.14\pm 0.04$ under continuous-wave excitation and $g^{\left(2\right)}\left(0\right)=0.21\pm 0.06$ under pulsed excitation. While these values satisfy the single-photon criterion of $g^{\left(2\right)}\left(0\right)<0.5$, they do not reach the ideal limit of $g^{\left(2\right)}\left(0\right)=0$. Furthermore, the lack of a nanocavity precluded the active control and enhancement of the spontaneous emission rate. As a result, subsequent studies therefore focused on integrating ${\mathrm{WSe}}_{2}$ single-photon emitters with resonant nanophotonic structures to enhance and control their emission. In particular, Luo et al. deterministically coupled strain-localised quantum emitters in monolayer ${\mathrm{WSe}}_{2}$ to plasmonic nanocavities formed by Au nanocubes, achieving Purcell factors as high as 551 and single-photon emission rates up to 42 MHz [5]. The Purcell enhancement did not affect the single-photon nature of the emission. One example showed $g^{\left(2\right)}\left(0\right)=0.15\pm 0.03$, which means a single-photon purity of about 85%. For 15 coupled emitters, the average $g^{\left(2\right)}\left(0\right)$ was 0.21. However, these measurements were performed at a cryogenic temperature of 3.8 K, and the plasmonic cavity had a low quality factor of Q∼8. Metallic losses also limited the quantum yield. To improve this further, it is necessary to reduce ohmic and non-radiative losses, obtain higher-Q resonances with good field confinement, improve the spatial and spectral overlap between the emitter and the field, and enable operation at higher or room temperature.

Reliable single-photon emission from TMDC monolayers at higher temperatures is important for their use in integrated quantum photonic technologies. To address this, Parto et al. used nanoscale strain engineering together with electron beam-induced defect engineering in monolayer ${\mathrm{WSe}}_{2}$. This approach produced site-controlled single-photon emitters with high fabrication yields and single-photon purities above 95% [95]. Photon antibunching was maintained up to 150 K, with $g^{\left(2\right)}\left(0\right)=0.27\pm 0.05$ at 150 K, corresponding to a single-photon purity of about 73%. This represents a significant improvement over native ${\mathrm{WSe}}_{2}$ emitters, which usually lose emission and single-photon purity above about 30 K. However, 150 K is still well below room temperature, indicating that further improvements in exciton confinement and radiative emission are required.

Localised excitons in monolayers such as ${\mathrm{WSe}}_{2}$ provide the microscopic basis for TMDC single-photon sources, as discussed above. However, their practical performance is limited by low extraction efficiency, long and variable emission lifetimes, spectral instability, and the random position and polarisation of the emitting dipole. As a result, integrating TMDC monolayers with metasurfaces provides a route by which to enhance radiative emission and improve photon extraction while potentially extending operation to elevated temperatures. In the following Section 5.1.1 and Section 5.1.2, we discuss how integrating TMDC monolayers with dielectric and plasmonic metasurfaces can improve the operating temperature and single-photon purity.

#### 5.1.1. SPSs by Dielectric Metasurfaces

As explained in the preceding section, dielectric metasurfaces can modify the local optical density of states and the far-field radiation pattern while avoiding the absorption losses associated with metallic structures in TMDC monolayer emitters. In contrast to the collective exciton–photon strong coupling discussed in Section 4.2, the relevant mechanism here is predominantly weak-coupling Purcell enhancement. The resonant dielectric environment increases the radiative decay rate and redirects the emitted photons, while antibunching originates from a localised exciton that can accommodate only one excitation at a time.

Early experiments established these functions using individual dielectric nanoantennas. Sortino et al. transferred monolayer ${\mathrm{WSe}}_{2}$ onto GaP dimer nanoantennas that provided both resonant optical enhancement and the local strain required to form confined excitons [96]. Compared with emitters formed on low-index ${\mathrm{SiO}}_{2}$ nanopillars, the GaP-coupled emitters exhibited a $10^{2}$–$10^{4}$ increase in photoluminescence intensity per unit excitation power. Their average quantum efficiency increased from 4±2% to 21±3%, with a maximum value of 86%. The enhanced efficiency enabled bright antibunched emission at excitation energies below 1 fJ per pulse. A related SiN dimer design was investigated by Azzam et al., who used the nanogap geometry to align the optical hotspot with the strain-localised ${\mathrm{WSe}}_{2}$ emitter [97]. The coupled emitters showed Purcell factors exceeding 20, sub-nanosecond decay components, and $g^{\left(2\right)}\left(0\right)=0.11$. The anisotropic strain and optical response of the dimers also produced polarisation extinction ratios as high as 94%. These studies show that a dielectric resonator can combine emitter formation, radiative rate enhancement, and polarisation control within one nanostructure.

More recently, Eng et al. described a metasurface-based approach in which a ${\mathrm{WSe}}_{2}$ monolayer was integrated with a periodic array of monocrystalline Si nanoantennas supporting a qBIC [98]. The broken-symmetry unit cell contained a 30 nm nanogap that produced a surface-accessible qBIC hotspot and also induced the local deformation of the ${\mathrm{WSe}}_{2}$ monolayer. When the emitter was coupled to the qBIC nanoantenna, it showed $g^{\left(2\right)}\left(0\right)=0.19\pm 0.02$ at 4 K, confirming that single-photon emission was preserved after integration with the dielectric structure. Its fast decay component decreased from 0.86±0.04 ns off the antenna to 0.082±0.005 ns on the antenna, corresponding to an effective Purcell factor of approximately 10. Here, the qBIC metasurface also altered the polarisation and momentum distribution of the emitted photons, with the on-antenna emitters being preferentially oriented in the direction of the polarisation of the qBIC mode, while the back-focal-plane measurements indicated directional emission.

Although the TMDC devices discussed above remain in the Purcell enhancement regime, carbon-related colour centres in few-layer hBN have enabled strong coupling between a two-dimensional single-photon emitter and a dielectric metasurface. This system exhibited Rabi splitting of approximately 4 meV and $g^{\left(2\right)}\left(0\right)=0.28$ at room temperature [99]. Extending this approach to localised excitons in TMDC monolayers remains an open experimental objective.

#### 5.1.2. SPSs by Plasmonic Metasurface

Plasmonic structures can enhance emission from localised TMDC quantum emitters through the Purcell effect and improve photon extraction. In this regime, photon antibunching originates from the individual emitter and does not require exciton–plasmon strong coupling. Neither the Purcell factor nor the cavity quality factor alone determines single-photon purity, which must be assessed through measurements of $g^{\left(2\right)}\left(0\right)$.

Photon blockade provides a different mechanism. Conventional blockade relies on an anharmonic excitation spectrum that spectrally suppresses access to higher excitation states. Unconventional blockade instead suppresses two-photon occupation through destructive interference between excitation pathways and can occur without spectrally resolved strong coupling. Consequently, collective exciton–plasmon anticrossing alone cannot establish either mechanism.

**Figure 7:**
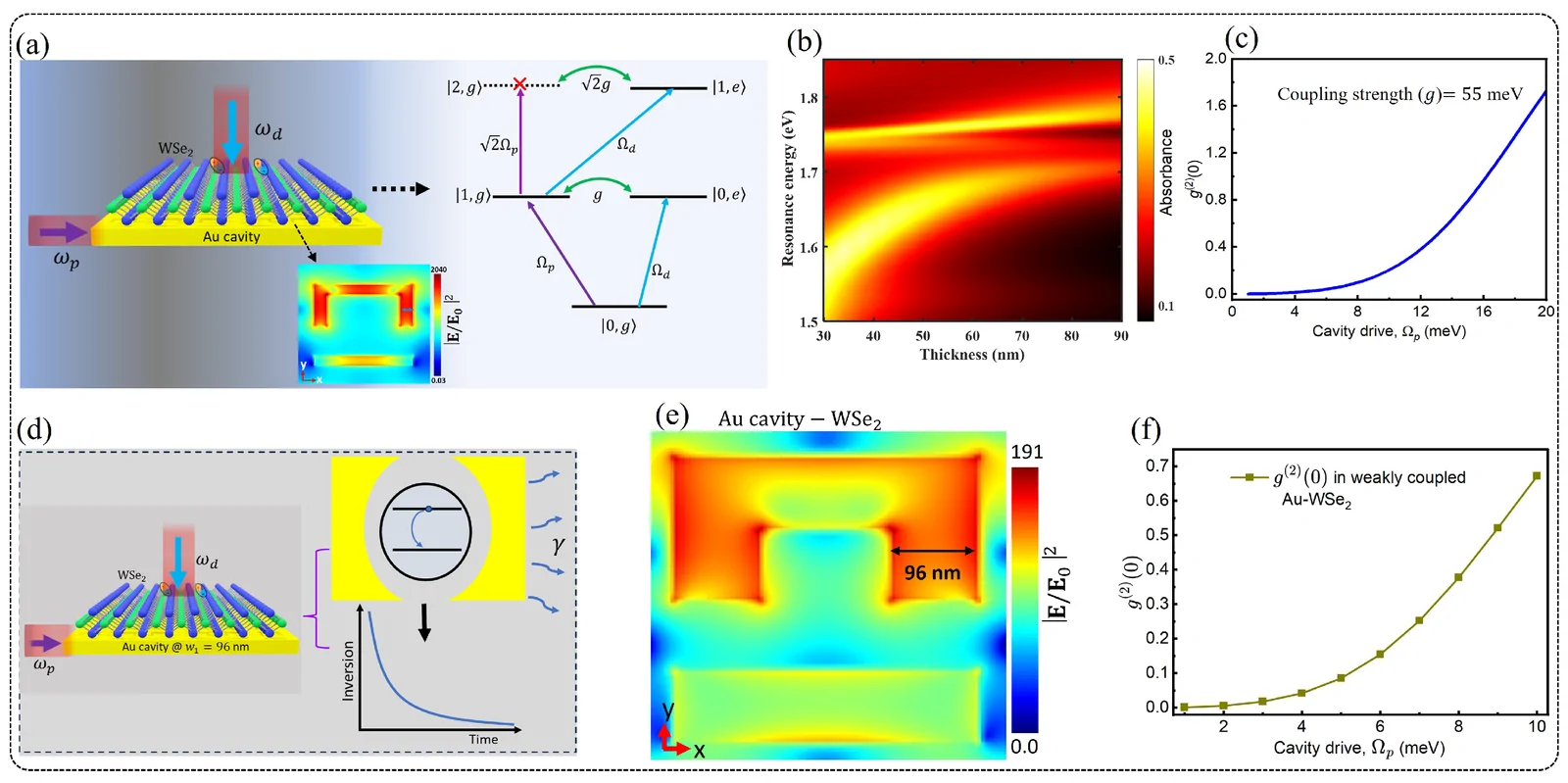
Comparison of single-photon emission purity in strongly and weakly coupled Au metasurface-${\mathrm{WSe}}_{2}$ monolayer systems. (**a**) Schematic of the strong coupling Au–${\mathrm{WSe}}_{2}$ system and Jaynes–Cummings energy levels for UnPB, where the coherent cavity and exciton drives induce destructive interference between two-photon excitation pathways. The inset shows the unit-cell $|{E/E}_{0}|\;=2040$. (**b**) Absorption spectrum mapping of Au–${\mathrm{WSe}}_{2}$ with varying Au thickness from 30 to 90 nm while keeping the other parameters constant ($W_{1}=39$ nm, $W_{2}=33$ nm, h=106 nm, g=106 nm, α=307 nm, and p=420 nm). Thus, it demonstrates strong coupling in Au–${\mathrm{WSe}}_{2}$ as a function of the Au thickness, with Rabi splitting of >100 meV. (**c**) $g^{\left(2\right)}\left(0\right)$ as a function of cavity drive strength $\Omega_{p}$ for the strongly coupled Au–${\mathrm{WSe}}_{2}$ system at g=55 meV; at $\Omega_{p}=0.5$ meV, $g^{\left(2\right)}\left(0\right)=1.64\times 10^{-4}$, corresponding to a single-photon purity of 99.98%. (**d**) Schematic of the Au–${\mathrm{WSe}}_{2}$ design and the corresponding spontaneous emission decay over time. (**e**) $|{E/E}_{0}|$ of the weakly coupled hybrid Au–${\mathrm{WSe}}_{2}$ structure, reaching ≈191 for the wider-gap geometry. (**f**) $g^{\left(2\right)}\left(0\right)$ versus cavity drive strength for the weak coupling UnPB configuration, for which the reported minimum is approximately $1.2\times 10^{-3}$. At low drive, the strongly coupled configuration reduces the residual two-photon probability by about 7.3 times. Reproduced from ref. [18] under CC BY 4.0.

Berhe et al. theoretically investigated an interference-based unconventional photon blockade scheme in a TMDC–plasmonic metasurface model [18]. Figure 7 summarises the calculated coupling and photon correlation properties. Here, the strongly coupled Au–${\mathrm{WSe}}_{2}$ configuration first exploits the geometrical tunability of the EQ-dominated plasmon resonance to achieve spectral matching with the ${\mathrm{WSe}}_{2}$ exciton (Figure 7a–c), after which the two-drive unconventional photon blockade scheme suppresses the two-photon excitation pathway (see Figure 7a,d). At a cavity drive strength of $\Omega_{p}=0.5$ meV, the strongly coupled system reaches $g^{\left(2\right)}\left(0\right)=1.64\times 10^{-4}$, corresponding to a single-photon purity of 99.98% [18]. In comparison, as shown in Figure 7d–f, increasing the metasurface gap reduces the field confinement and moves the Au–${\mathrm{WSe}}_{2}$ system into the weak coupling regime, where $g^{\left(2\right)}\left(0\right)\approx 1.2\times 10^{-3}$ is obtained under the same interference-based scheme. These calculations predict pronounced antibunching under the specified two-drive interference scheme in both coupling regimes. The lower calculated $g^{\left(2\right)}\left(0\right)$ in the strongly coupled configuration is therefore a result of the particular model and driving conditions, rather than evidence that collective strong coupling alone produces single photons. Although the numerical results demonstrate high single-photon purity at room temperature, experimental validation is required to confirm single-emitter nonlinear strong coupling in TMDC–metasurface systems under these conditions.

The purity of the photons emitted can be greatly improved by designing a suitable photonic/plasmonic cavity coupled with QEs to enhance light–matter interaction. Furthermore, a hybrid structure comprising plasmonic nanocavities and TMDC monolayers is promising for designing and fabricating miniaturised and integrable SPEs. In this context, the gold (Au) cavity plays a crucial role because of its stability and ability to confine electromagnetic fields within a small volume, which directly contributes to high purity.

Table 3 shows summary of experimental demonstrations and numerical modelings of single-photon emission from monolayers integrated with plasmonic and dielectric metasurfaces/nanostructures.

Having considered TMDC–metasurface systems for single-photon emission (see Section 5.1), the discussion is next extended to the higher-excitation regime required for lasing. Single-photon sources operate at low excitation levels, where emission from an individual localised excitonic state is isolated and antibunching can be observed. As a result, in Section 5.2, we discuss lasing in TMDC monolayers coupled with metasurfaces; as the optical pump density increases, the system enters a many-exciton regime in which stimulated scattering and stimulated emission become increasingly important.

### 5.2. Nanoscale Lasers

Conventional photon lasing and polariton lasing require different physical conditions. Photon lasing occurs when stimulated emission provides a sufficient gain to compensate for the cavity losses and does not require exciton–photon strong coupling. Polariton lasing involves stimulated scattering into a polariton mode while the system remains strongly coupled. A nonlinear increase in emission and linewidth narrowing can occur in both regimes; identifying polariton lasing additionally requires evidence that the emission originates from a polariton branch and that strong coupling persists across the threshold, together with the emergence of optical coherence.

**Figure 8:**
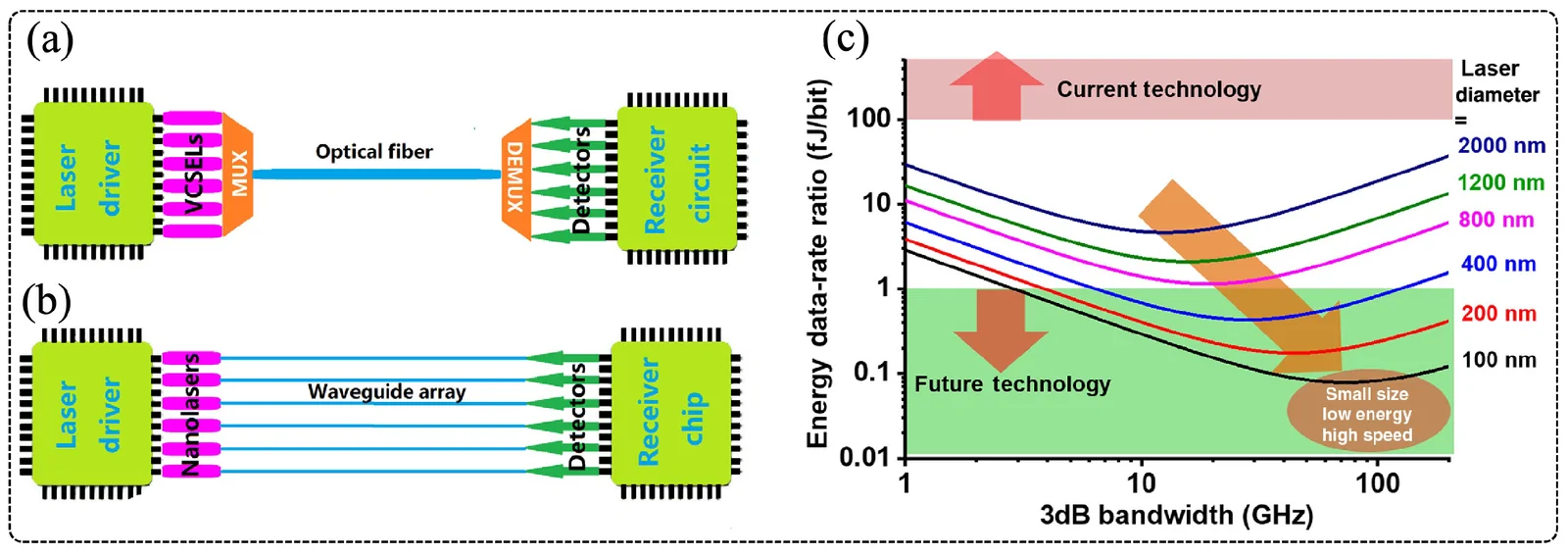
(**a**) Schematic of a present-day off-chip optical interconnect, in which an array of vertical cavity surface-emitting lasers (VCSELs) driven by a laser driver is multiplexed into an optical fibre and demultiplexed onto detectors at the receiver circuit. (**b**) Target on-chip interconnect, in which an array of nanolasers is edge-coupled directly to an on-chip waveguide array, setting the size, energy, and areal density requirements that the source must satisfy. (**c**) Energy–data rate ratio (EDR, in fJ/bit) versus 3 dB modulation bandwidth for cylindrical semiconductor lasers with diameters of 100–2000nm. Each diameter exhibits an optimum bandwidth at which the EDR is minimised, and only diameters of a few hundred nanometres or less reach the sub-fJ/bit regime. The arrow indicates the direction of decreasing size and energy consumption and increasing speed pursued in nanolaser research. Reproduced from ref. [102] under the CC BY 4.0 licence.

Nanoscale lasers are motivated by the need to integrate compact, low-energy light sources with photonic circuits [102]. Figure 8 illustrates the transition from fibre-coupled VCSEL arrays to on-chip sources and the associated size and energy constraints. For the metasurface–TMDC systems considered here, the relevant question is whether an atomically thin gain medium can provide a sufficient modal gain to overcome cavity loss while retaining feedback and efficient out-coupling.

Dielectric cavities can provide low-loss optical feedback, while plasmonic modes can concentrate the field within a much smaller volume near the TMDC monolayer. Early plasmonic nanolasers based on other gain materials demonstrated the potential of subwavelength optical confinement [103,104], but their lasing results cannot be taken as evidence for metasurface–TMDC devices. The latter must be assessed using their own gains, optical losses, emission thresholds, linewidths, and coherence measurements. The following subsections therefore focus on reported TMDC monolayer systems.

Subsequently, alongside cavity design, nanoscale lasing requires a gain medium that provides a strong optical gain without sacrificing the nanoscale footprint. Monolayer TMDCs combine an atomically thin active volume with strong excitonic transitions and large exciton binding energies, allowing the excitonic optical response to persist at room temperature and offering a route toward gains without requiring a large active material volume (see Section 2). Their atomic-scale thickness simultaneously minimises the active volume and facilitates integration with photonic and plasmonic platforms [102,105]. The metasurface confines the optical mode and enhances the local field, whereas the TMDC monolayer concentrates the optical oscillator strength within a minimal active volume. Their combination offers a route toward an ultracompact laser but does not by itself guarantee lasing, since the optical gain must exceed the combined radiative, absorptive, and non-radiative losses [23,102]. This requirement motivates the engineering of metasurface resonances with a favourable $Q/V_{\mathrm{eff}}$ and strong spatial and polarisation overlap with the TMDC monolayer.

The emphasis on plasmonic metasurfaces for extreme miniaturisation does not diminish the importance of dielectric metasurfaces. Dielectric metasurfaces can provide excellent room-temperature lasing performance by exploiting high-*Q* guided-mode or qBIC resonances, although their optical modes generally extend over larger lateral dimensions than deeply confined plasmonic modes. Accordingly, Section 5.2.1 and Section 5.2.2 discuss recent advances in dielectric and plasmonic metasurface lasers, respectively, and compare their different trade-offs between optical loss, mode confinement, and true lasing in TMDC monolayers.

#### 5.2.1. Lasing by Dielectric Metasurfaces

The dielectric metasurfaces discussed in Section 4.2 provide low-loss resonances suitable for exciton–photon strong coupling. However, linear anticrossing does not establish polariton lasing. In an initially strongly coupled device, exciton saturation or bleaching can destroy strong coupling at high pump densities, allowing a transition to conventional photon lasing. Other devices may operate as photon lasers without first entering strong coupling. Polariton lasing must therefore be identified by the persistence of strong coupling across the emission threshold and coherent emission from the polariton mode. An interaction-induced energy shift can provide supporting evidence but is not sufficient on its own.

**Figure 9:**
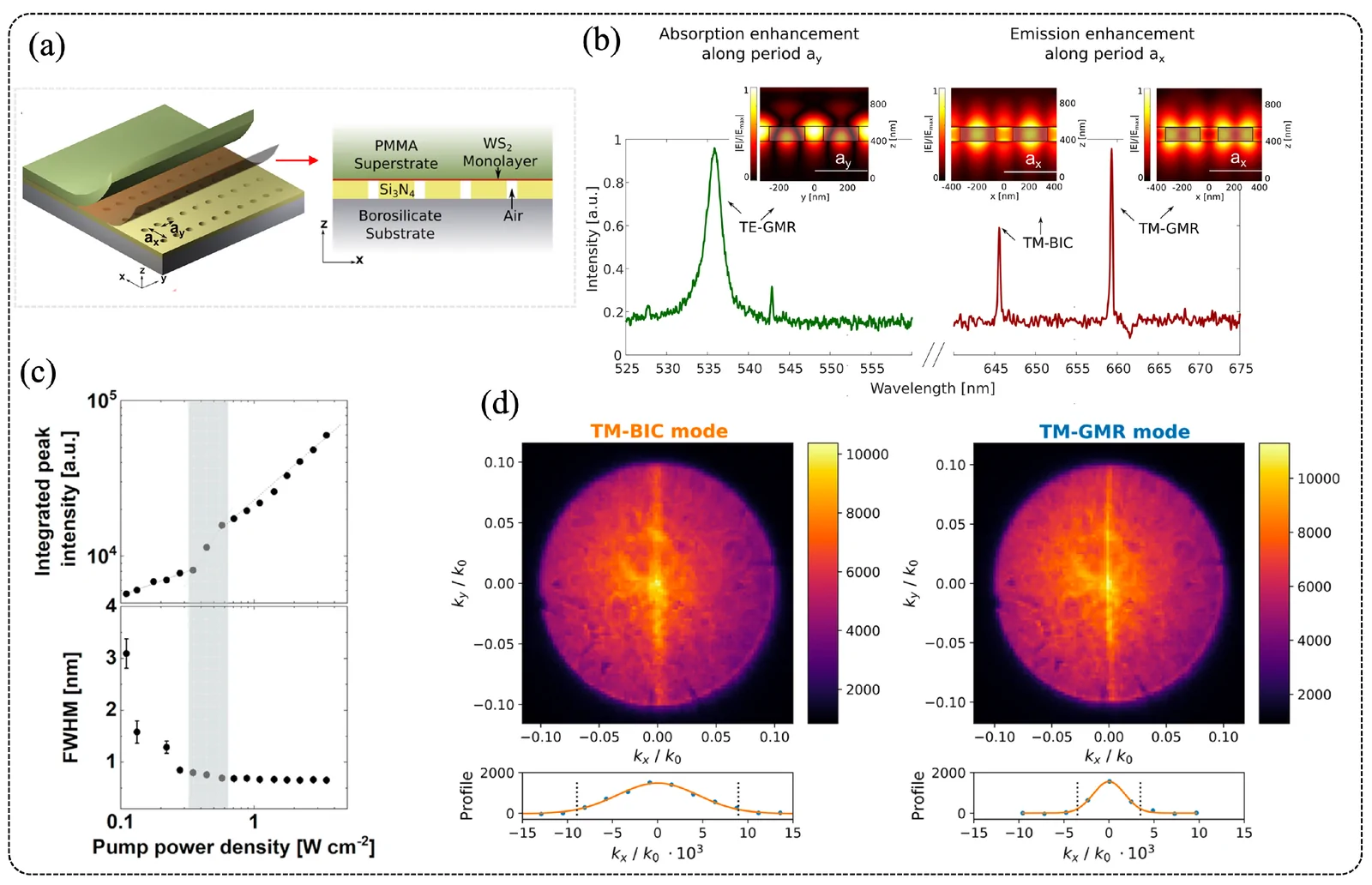
Room-temperature monolayer ${\mathrm{WS}}_{2}$ lasing enabled by a dual-resonance dielectric metasurface. (**a**) A 3D schematic and its corresponding 2D side view of the laser architecture comprising a ${\mathrm{WS}}_{2}$ monolayer integrated with a rectangular ${\mathrm{Si}}_{3}$$N_{4}$ nanohole array metasurface. (**b**) Experimental optical response of the dual-resonance metasurface, showing a TE-GMR near the pump wavelength and high-Q TM-GMR and symmetry-protected BIC (TM-BIC) modes near the ${\mathrm{WS}}_{2}$ emission wavelength. (**c**) Pump power-dependent laser emission showing the nonlinear input–output response and linewidth narrowing across the lasing threshold. (**d**) Far-field radiation patterns of the TM-BIC and TM-GMR lasing modes, demonstrating the directional emission imposed by the metasurface dispersion. Adopted from ref. [106] under CC BY 4.0.

Recent dielectric metasurface lasers illustrate both the progress toward this objective and the distinction between photon and polariton lasing. Barth et al. demonstrated room-temperature lasing from a wafer-scale MOCVD-grown ${\mathrm{WS}}_{2}$ monolayer integrated with a dual-resonance dielectric metasurface (see Figure 9a for the design) [106]. Here, as can be seen in Figure 9b, the rectangular lattice simultaneously enhanced absorption at the pump wavelength and provided high-*Q* resonances at the emission wavelength. Specifically, a relatively low-*Q* TE GMR was employed near the 532 nm pump wavelength. In contrast, TM-GMR and symmetry-protected TM-BIC modes with Q∼3000 provided resonant feedback near the ${\mathrm{WS}}_{2}$ emission wavelength. Lasing occurred at a threshold below $1\;W\;{\mathrm{cm}}^{-2}$, with the nonlinear input–output response and linewidth narrowing providing clear evidence of the lasing transition (see Figure 9c), while, as can be seen in Figure 9d, the corresponding far-field measurements revealed directional emission associated with the TM-BIC and TM-GMR lasing modes. The device also exhibited a measured spatial coherence length exceeding 30μm. These findings show that dielectric metasurfaces can overcome the limited absorption and gain volume inherent to atomically thin active media. However, the emission was identified as conventional photon lasing, as the anticrossing in the hybrid dielectric metasurface–${\mathrm{WS}}_{2}$ monolayer did not show strong coupling. Consequently, the device should be classified as a monolayer–metasurface laser rather than a polariton laser.

These results reveal an important distinction between the current capabilities of TMDC–dielectric metasurface designs and the requirements for a polariton laser. Strong coupling and room-temperature polariton formation have been demonstrated in dielectric photonic structures, while coherent polariton emission has been observed in TMDC nanocavities. In parallel, dielectric metasurfaces have enabled low-threshold photon lasing from large-area TMDC monolayers. The remaining challenge is therefore to combine these capabilities in a single device and demonstrate stimulated polariton occupation while maintaining strong coupling across the nonlinear emission threshold.

#### 5.2.2. Lasing by Plasmonic Metasurfaces

Plasmonic metasurfaces provide a complementary route to nanoscale lasing. As discussed in Section 3 and Section 4.3, their principal advantage is the confinement of electromagnetic energy within deeply subwavelength mode volumes. Although the active material was not a TMDC monolayer, Ramezani et al. achieved room-temperature lasing by arranging silver nanoantennas in a periodic pattern and strongly coupling them to organic excitons [69]. The interaction between localised plasmon resonances and Rayleigh anomalies produces narrow SLRs, while mixing with molecular excitons causes Rabi splitting of about 200 meV. Lasing is demonstrated through a dark quadrupolar plasmonic mode with relatively low radiative loss, which promotes polariton accumulation and reduces the lasing threshold. Still, using organic dye molecules for gain can lead to concentration quenching and photodegradation under strong optical pumping, which may reduce the long-term stability and efficiency of plasmon–exciton–polariton lasers. Realising a nanoscale laser based on this concept with a TMDC monolayer as the gain medium therefore remains an important objective [1].

**Figure 10:**
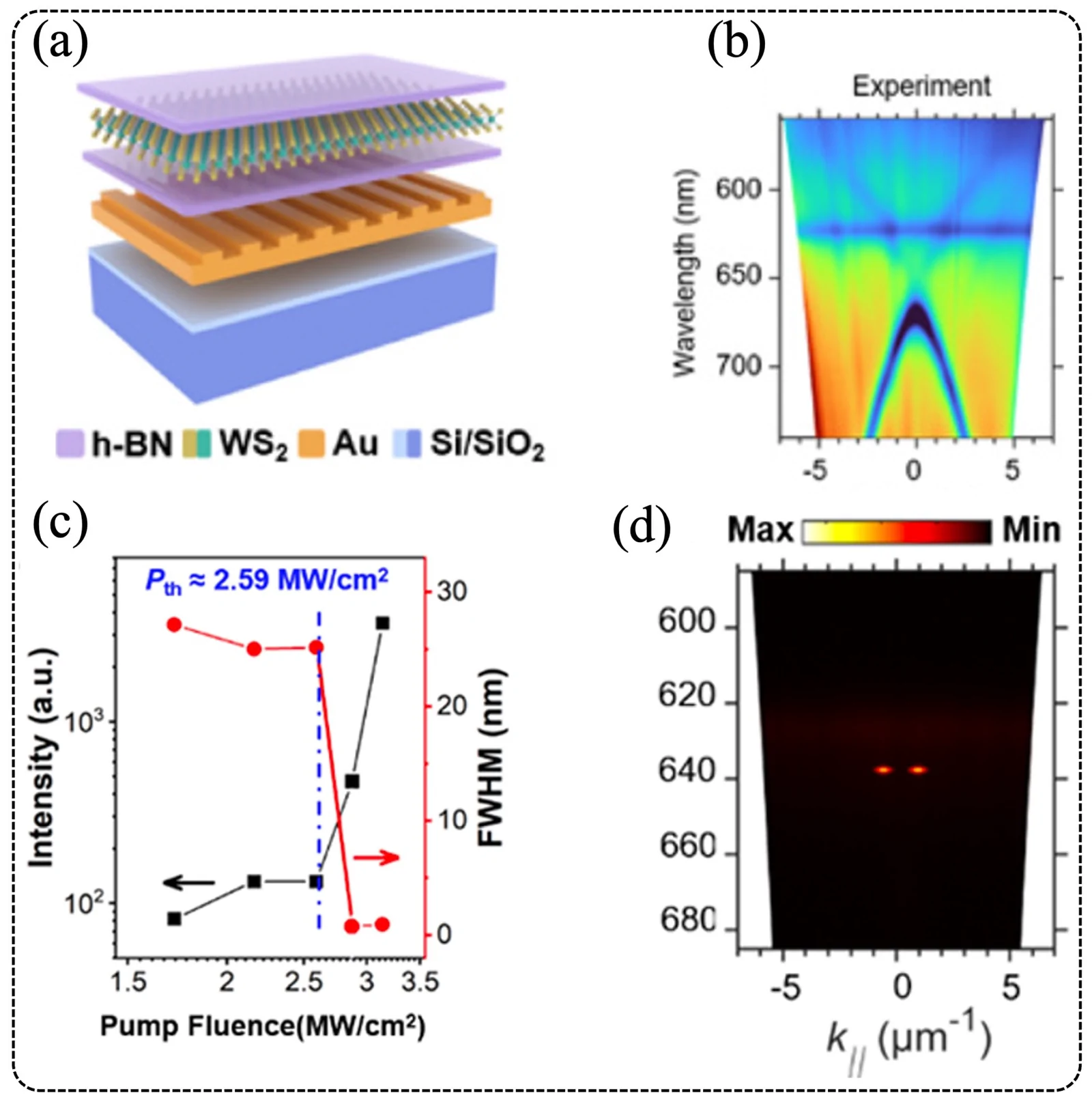
Room-temperature stimulated emission from a monolayer ${\mathrm{WS}}_{2}$–plasmonic quasi-BIC heterostructure. (**a**) Schematic of the hBN-encapsulated monolayer ${\mathrm{WS}}_{2}$ on a plasmonic grating milled into a single-crystalline Au microplate. (**b**) Measured momentum-resolved reflection spectrum of the optimised heterostructure (508 nm period, 52 nm grating height, 28.5 nm h-BN), showing the interruption of the SPP dispersion at the symmetry-protected plasmonic BIC at the Γ point. (**c**) Emission intensity and FWHM at 637 nm versus pump fluence, showing the threshold at approximately $2.59\;\mathrm{MW}\;{\mathrm{cm}}^{-2}$ and the narrowing of the emission peak from ∼25 nm to ∼0.7 nm. (**d**) Momentum-resolved photoluminescence above threshold ($1.2P_{\mathrm{th}}$), showing spectrally and angularly localised emission at λ≈637 nm and $k_{\|}\approx \pm 0.8\;\mu m^{-1}$. Figure (**a**–**d**) adapted with permission from [24] © Optical Society of America.

In this regard, Yang et al. recently demonstrated an important experimental step toward room-temperature plasmon lasing in a plasmonic grating–TMDC monolayer, although the system was not shown to operate as a polariton laser [24]. As can be seen in Figure 10a, the CVD-grown monolayer ${\mathrm{WS}}_{2}$ was encapsulated in exfoliated h-BN and integrated with a focused ion beam-milled plasmonic grating on a single-crystalline Au microplate. The periodic Au grating supports SPP modes whose dispersion can be engineered through the grating geometry. The optimisation of the structure (508 nm period, 52 nm grating height, and 28.5 nm h-BN thickness) additionally produced a symmetry-protected plasmonic bound state in the continuum (BIC) near the Γ point, manifested as an interruption in the SPP dispersion near 640 nm (see Figure 10b). The metagrating exhibited a maximum measured quality factor of Q≈54, while simulations predicted Q>120 closer to the Γ point. As a result, as shown in Figure 10c, under 532 nm nanosecond pulsed excitation, the hybrid structure exhibited a pronounced threshold-like transition at a peak pump intensity of approximately $2.59\;\mathrm{MW}\;{\mathrm{cm}}^{-2}$, accompanied by a rapid increase in emission intensity and the narrowing of the emission peak from approximately 25 nm below the threshold to 0.7 nm above it. Furthermore, momentum-resolved measurements revealed that the initially broad, angularly uniform spontaneous emission evolved above the threshold into two spectrally and angularly localised spots at λ≈637 nm and $k_{\|}\approx \pm 0.8\;\mu m^{-1}$, corresponding to directional emission at approximately ±4.65° (see Figure 10d). However, even though the threshold-like nonlinear response, strong spectral narrowing, and momentum-selective emission provided compelling signatures of stimulated emission, neither optical coherence nor photon statistics were measured, and no output power was reported. As a result, these quantum threshold conditions could not be evaluated [107].

To the best of our knowledge, true lasing in TMDC monolayer–plasmonic metasurfaces has not been unambiguously demonstrated experimentally. Demonstrating room-temperature lasing in a plasmonic metasurface–TMDC monolayer requires the modal gain to exceed the total cavity loss, $\Gamma g_{\mathrm{TMDC}}>\alpha_{\mathrm{tot}}$, where $\alpha_{\mathrm{tot}}=\alpha_{\mathrm{abs}}+\alpha_{\mathrm{rad}}+\alpha_{\mathrm{scat}}$ represents the combined absorptive (primarily ohmic), radiative, and scattering losses, respectively. This condition must be satisfied while maintaining sufficient optical confinement, feedback, and controlled out-coupling. Because the threshold depends on the balance between gain and loss rather than the quality factor alone, a plasmonic design must compensate for its intrinsically higher absorptive loss through strong field confinement and enhanced overlap with the atomically thin gain medium. This remains a major challenge compared with dielectric implementations: room-temperature ${\mathrm{WS}}_{2}$ monolayer lasers based on high-*Q* photonic resonances have already been demonstrated [106,108].

A promising strategy is therefore to combine the field concentration of localised plasmonic modes with the suppressed radiative loss of collective resonances such as SLR, Fano, or quasi-BIC modes, while incorporating a second resonance at the pump wavelength to enhance absorption in the sub-nanometre-thick gain layer. Additionally, the fabrication quality is equally important: low-loss, low-roughness Au or Ag should be patterned with nanometre-scale dimensional control, followed by the integration of a high-quality TMDC monolayer with a thin h-BN or dielectric spacer to suppress metal-induced non-radiative quenching without sacrificing near-field overlap. Chemical passivation can further suppress defect-mediated non-radiative recombination and substantially increase the photoluminescence quantum yield of monolayer TMDCs [52]. Finally, enhanced photoluminescence alone is insufficient to establish lasing. A convincing demonstration should show a reproducible nonlinear input–output threshold, linewidth narrowing, emission locked to the designed cavity mode, and polarisation-resolved and directional far-field emission, together with optical coherence measurements and sufficient output power information to assess the laser performance [107]. Control measurements on the bare monolayer, unloaded metasurface, and spectrally detuned devices are additionally desirable to establish that the observed transition originates from the engineered plasmonic cavity.

Such confinement increases the local coupling strength and reduces the area of high-quality TMDC material that must overlap with the resonant mode. This feature is particularly relevant to mechanically exfoliated monolayers, whose lateral dimensions and spatial uniformity often limit their integration with extended dielectric resonances. The same confinement, however, increases absorption in the metal and introduces local heating, so strong coupling does not necessarily translate into a sufficiently long polariton lifetime for stimulated scattering and macroscopic occupation.

As discussed above, room-temperature polariton condensation has not yet been demonstrated in a strongly coupled metasurface–TMDC-monolayer system. However, Sung et al. demonstrated room-temperature polariton condensation in a BIC metasurface patterned directly into multilayer ${\mathrm{WS}}_{2}$ (∼50 nm), achieving Rabi splitting of 294 meV. This self-hybridised structure combines strong exciton–photon coupling, suppressed radiative losses, and macroscopic spatial coherence, highlighting the potential of BIC-based TMDC cavities for polariton lasing [109]. Nevertheless, these results should not be taken as evidence that equivalent performance can be achieved in a TMDC monolayer–dielectric-metasurface system. Instead, the study provides a useful design benchmark: a successful monolayer device must simultaneously preserve strong coupling, provide an efficient relaxation pathway into the selected polariton state, and maintain sufficiently low total losses to reach the nonlinear threshold.

As discussed in Section 5.2.1 and Section 5.2.2 and in Table 4, although polariton lasing has been demonstrated in microcavities incorporating multiple TMDC monolayers separated by hBN [110], it has not yet been demonstrated in metasurface–TMDC systems. To demonstrate this in a dielectric metasurface with multiple TMDC monolayers separated by hBN and with a single monolayer on a plasmonic metasurface, a high-quality fabricated metasurface with a sufficiently high Q factor is required. Therefore, Section 6 presents experimental fabrication routes and the structural factors that influence the optical loss, exciton quality, and field overlap.

## 6. Experimental Fabrication of Metasurfaces and Integration with TMDC Monolayers

**Figure 11:**
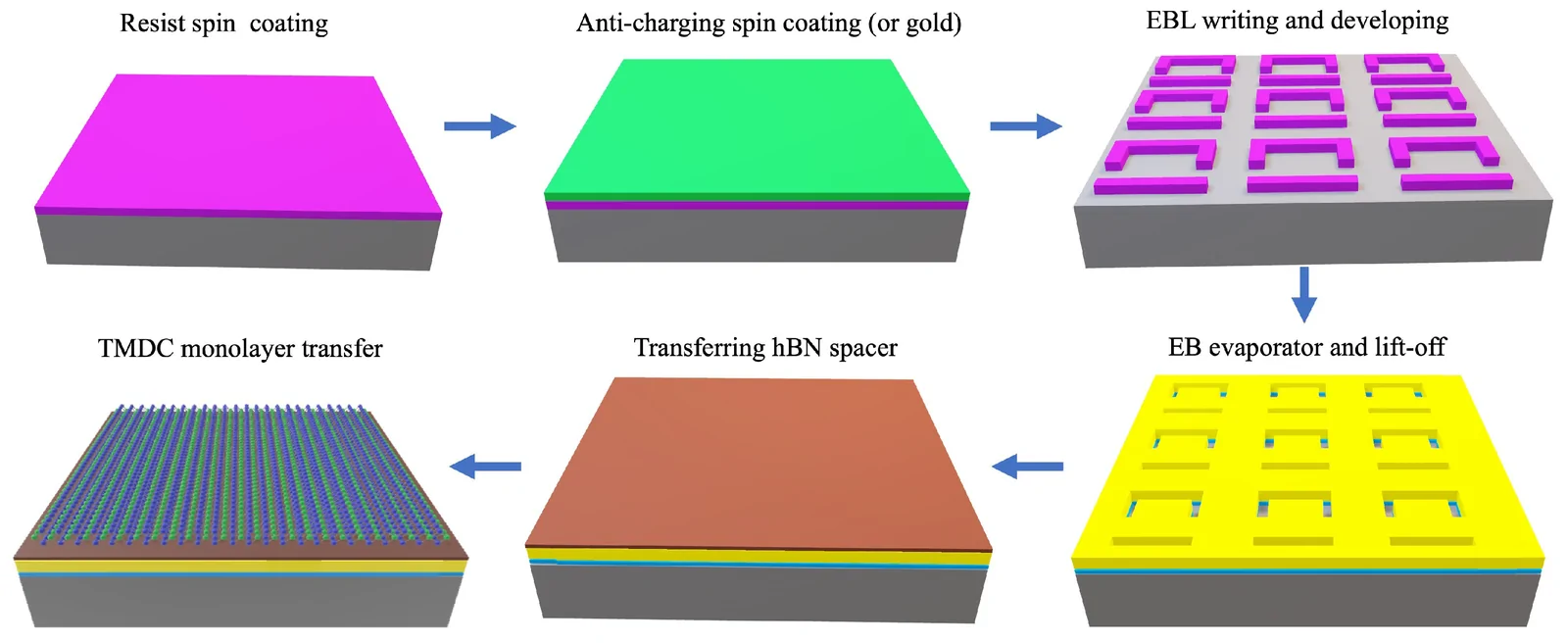
Representative fabrication and integration sequence for a plasmonic metasurface–TMDC monolayer hybrid. Electron beam lithography defines the metallic pattern, followed by metal deposition and lift-off. Finally, a thin spacer is deposited, or hBN is transferred, and the TMDC monolayer is then integrated with the resonator.

The fabrication of metasurface–TMDC hybrids commonly combines the lithographic patterning of dielectric or metallic resonators with the transfer of an exfoliated or grown monolayer [111]. Figure 11 illustrates a representative electron beam lithography (EBL) and transfer sequence for a plasmonic structure. To interpret strong coupling results, the key fabrication parameters are the resonator dimensions and roughness, which affect the optical loss and *Q*; monolayer damage and contamination, which affect the exciton linewidth; and the position and thickness of any spacer, which balance near-field overlap against metal-induced quenching. As a result, these parameters should be considered alongside the experimentally measured optical and excitonic spectra.

Following a fabrication procedure similar to that shown in Figure 11, Gherman et al. fabricated periodic Au nanoparticle-over-mirror arrays using EBL, Au deposition, and lift-off [112]. The structures supported propagating SPPs coupled to localised plasmonic modes and ${\mathrm{WSe}}_{2}$ excitons, demonstrating strong coupling. Although the reported resonance is not explicitly an SLR, this approach shows that periodic plasmonic arrays can be fabricated for SLR engineering. The reported work used a 17 nm ${\mathrm{Al}}_{2}$$O_{3}$ spacer, highlighting the importance of controlling the spacer thickness to balance plasmonic coupling, field confinement, and exciton quenching.

A distinct fabrication route is to pattern the excitonic material itself: Sung et al. etched a BIC-supporting nanohole array directly into multilayer ${\mathrm{WS}}_{2}$ [109]. Because this structure uses an approximately 50 nm thick multilayer rather than a transferred monolayer, its fabrication approach and condensation result should be identified separately from those of monolayer-on-metasurface devices.

## 7. Emission Control Using Dielectric Metasurfaces

The single-photon sources (SPSs) and nanoscale lasers discussed in Section 5.1 and Section 5.2 require an optical interface to couple locally generated photons into useful propagating channels. Even with strong optical confinement, efficient photon collection is not guaranteed, since nanoscale emitters can radiate over a wide range of angles, while coupling into fibres or photonic circuits may be limited by substrate trapping and spatial or polarisation mismatches. To address these limitations, a separately designed, low-loss dielectric metasurface or metalens placed on the output side can impose a spatially varying phase profile and, through anisotropic meta-atoms, a polarisation-dependent response [113]. Appropriate profiles enable collection, collimation, focusing, beam steering, and vortex generation. As shown in Figure 1, the metasurface–TMDC hybrid provides the active light–matter interaction and emission, while the dielectric ${\mathrm{Si}}_{3}$$N_{4}$ manipulates the emitted photons. For instance, Berhe et. al. showed an all-pass ${\mathrm{Si}}_{3}$$N_{4}$ metasurface that combined the functions of a metalens and a spiral phase plate in one device, allowing the highly efficient focusing of optical vortex beams. At a wavelength of 1100 nm, the metasurface produced vortex beams with topological charges as high as ℓ=5 and achieved focusing efficiency of 90.7% while keeping high OAM purity, because of the near-unity transmission and the full 2π phase control made possible by the ${\mathrm{Si}}_{3}$$N_{4}$ nanopillars [27]. Nevertheless, in order to apply this multifunctional approach to the visible spectral range, it would be necessary to proportionally reduce the lateral dimensions and the period of the ${\mathrm{Si}}_{3}$$N_{4}$ meta-atoms and to re-optimise their height and radius in order to regain high transmission and complete 2π phase coverage at the target TMDC emission wavelength. When applied to the TMDC–metasurface quantum/coherent light sources mentioned above, a visible ${\mathrm{Si}}_{3}$$N_{4}$ meta-optic could thus simultaneously collect and focus the emitted light.

In the SPS system, et al. experimentally demonstrated a multifunctional dielectric metalens for manipulating room-temperature single-photon emission from hBN defects in the visible spectral range. The metalens was designed for visible emission at approximately 610 nm using low-loss a-Si:H meta-atoms and achieved a high numerical aperture of 0.869, with average meta-atom transmission of approximately 94% [114]. Importantly, the metalens not only collected and collimated the single-photon emission but also controlled its directionality and polarisation. By incorporating helical phase profiles into the same metalens, they further generated OAM states with topological charges of ℓ=3 and ℓ=−1 for two orthogonal polarisation channels. The antibunching behaviour was preserved after wavefront manipulation, confirming that the spatial and OAM properties of the emitted photons could be tailored without destroying their single-photon character.

The passive metasurface can also manipulate the coherent light source. Xie et al. demonstrated a non-intrusive approach for manipulating the output of a VCSEL by monolithically integrating a dielectric metasurface onto the back-emitting surface of the device [115]. Although a conventional VCSEL provides compact and efficient coherent light generation, its output remains strongly divergent; in this work, the bare VCSEL exhibited a divergence angle of approximately 30.4°. The integrated metasurface addressed this limitation by imposing a spatially varying phase on the emitted wavefront after the light had left the laser cavity, rather than modifying the lasing mechanism itself. The required phase profile for collimation was expressed as

(18) $$\phi_{\mathrm{collimator}}\left(x,y\right)=2\pi -\frac{2\pi n}{\lambda }\left(\sqrt{x^{2}+y^{2}+f^{2}}-f\right),$$

where λ is the lasing wavelength, *n* is the refractive index of the substrate, and *f* is the focal length. The phase distribution counteracts the curved wavefront that builds up as light from the VCSEL travels through the substrate, transforming the highly divergent output into a nearly collimated beam. In experiments, GaAs nanopillars of various diameters were arranged according to this phase profile, with the nanopillar diameters determining the local phase delay. When an optimised focal length of f=630 μm was used, the metasurface reduced the divergence angle from about 30.4° for the bare VCSEL to just 0.83°, while achieving approximately 80% transmission and 57% collimation efficiency. In addition to simple collimation, further phase profiles were added to $\phi_{\mathrm{collimator}}$ in order to convert the same VCSEL output into non-diffracting Bessel beams, OAM-carrying vortex beams, and laser beams that could be directionally steered. The study thus demonstrates that a dielectric metasurface can provide wavefront control that is inherently missing from a conventional VCSEL while having only a small effect on the main light generation mechanism. Similarly, by designing and fabricating a dielectric metasurface at the exciton resonances of the TMDC monolayers, the emitted light can be manipulated effectively.

## 8. Conclusions and Outlook

### 8.1. Conclusions

In this review, we have examined the theory, design, and experimental realisation of strong light–matter coupling in metasurface–TMDC monolayer systems, together with the two applications that follow most directly from it: single-photon emission and lasing. We began with the case for TMDC monolayers as the excitonic component has high oscillator strength, exciton binding energies of several hundred meV that keep excitons intact at room temperature, and a 0.7 nm active thickness that permits placement at a field antinode, before turning to the photonic component, where the relevant figure of merit is Q/V. It is important to consider separately what each factor influences: the vacuum Rabi splitting scales as $\hbar \Omega \propto \sqrt{N/V}$ and is unaffected by the quality factor, whereas the quality factor determines whether the splitting can be resolved, via the strong coupling condition $\hbar \Omega >(\gamma_{\mathrm{exc}}+\kappa_{\mathrm{cav}})/2$. However, the reported Rabi splittings cannot be compared directly without accounting for differences in the TMDC material, oscillator strength, exciton and cavity linewidths, field overlap, and operating temperature. A consistent comparison should therefore consider the coupling strength, linewidths, cooperativity, and mode overlap, while distinguishing numerical modelings from experimental measurements. This difference underpins the main comparison in this review.

Plasmonic metasurfaces achieve a value of ℏΩ greater than 100 meV, whereas their dielectric counterparts typically have a value of less than about 65 meV. This is not because of their low quality factors but because the very small mode volumes and strong in-plane field concentration at the metal surface overcome the vanishingly small spatial overlap available to a monolayer. Dielectric metasurfaces exhibit the opposite compromise, giving up field concentration to suppress radiative losses and achieving quality factors that are two orders of magnitude higher. This trade-off is directly reflected in device performance investigations. For single-photon emission, experimental demonstrations based on localised TMDC emitters must be distinguished from theoretical photon blockade schemes. Purcell enhancement can improve the emitter brightness without requiring strong coupling, whereas conventional photon blockade requires a sufficiently anharmonic excitation spectrum. The interference-based blockade predictions discussed here do not establish experimental single-emitter nonlinear strong coupling. For lasing, polariton lasing from a TMDC monolayer has been established in planar microcavities using hBN as a spacer with multiple monolayers. However, to the best of our knowledge, in a metasurface of either kind, the dielectric route is limited by weak spatial overlap and by a reservoir density that cannot be raised freely, since exciton–exciton annihilation and the Mott transition clamp the achievable gain well below what the huge oscillator strengths might suggest. Additionally, the plasmonic route is limited by photon lifetimes on the order of tens of femtoseconds, far shorter than the picosecond timescales on which polaritons relax. Photon lasing has also been reported in both systems, but an unambiguous demonstration of the effect in plasmonic metasurfaces, accompanied by the measured output power, first-order coherence, and photon statistics, has still not been achieved; the low Q factor may well be the key factor. We have therefore set out the experimental conditions that such a demonstration must satisfy, together with a clean-room fabrication route for high-*Q* plasmonic metasurfaces based on SLR or quasi-BIC designs. Taken together, these results indicate that metasurface-TMDC monolayer systems are approaching, but have not yet reached, the regime in which compact, ultrafast, and energy-efficient quantum nanophotonic devices become practical. Finally, we summarise low-loss dielectric metasurfaces for efficiently manipulating and collimating the generated light without changing its properties. Furthermore, we provide an outlook for developing compact, directional quantum and coherent light source designs.

### 8.2. Outlook

1. Directional single-photon emission using chiral plasmonic metasurfaces

Beyond enhancing spontaneous emission, plasmonic metasurfaces can engineer the polarisation and propagation directions of the photons emitted by monolayer TMDCs. Delocalised excitons provide a natural starting point, since the inequivalent *K* and $K^{\prime }$ valleys couple selectively to $\sigma^{+}$ and $\sigma^{-}$ circular polarisations, enabling the valley pseudospin to be interfaced with the spin and momentum of light. This principle has been demonstrated at the collective exciton–polariton level by Lee et al., who coupled monolayer ${\mathrm{WS}}_{2}$ to a plasmonic nanogroove metasurface and observed a valley Hall effect in which opposite circular polarisation components were routed in opposite directions through the spin–momentum locking of the plasmonic mode [116]. The nanogroove array is not geometrically chiral, and the directionality arises from the transverse optical spin of the surface plasmon modes, showing that plasmonic nanostructures can convert an internal polarisation property into directional optical transport. Monolayer TMDCs offer a further geometrical advantage: their exposed, atomically thin active layer sits directly within the plasmonic near-field, so the strain, field confinement, and emitter position can be engineered within one planar architecture. Extending this concept from delocalised excitons to localised emitters points toward directional, spin-encoded single-photon sources, although the valley selection rules of free excitons should not be assumed to survive localisation: Yang et al. showed that neutral quantum emitters in monolayer ${\mathrm{WSe}}_{2}$ coupled to chiral plasmonic nanocavities can emit circularly polarised single-photons. Interestingly, the helicity of the emitted photons was mainly determined by the handedness of the nanocavity rather than by the intrinsic valley-selective recombination of ${\mathrm{WSe}}_{2}$ [117]. Similarly, Lee et al. demonstrated spin angular momentum-encoded emission from strained ${\mathrm{WSe}}_{2}$ coupled to chiral Au nanoparticles, achieving antibunched emission with $g^{\left(2\right)}\left(0\right)∼0.3$ together with circular polarisation at a zero magnetic field [118]. Together, these studies offer a practical route to controlling single-photon emission from TMDCs with chiral plasmonic structures. Strong nanoscale field confinement enhances the interaction with a localised emitter, while structural chirality and spin–momentum coupling set the helicity and propagation direction of the emitted photons. A single chiral metasurface could therefore combine single-photon generation, Purcell enhancement, polarisation control, and directional emission, acting as an engineered optical environment rather than merely an extraction structure.

2. Highly directional nanoscale metasurface–TMDC monolayer lasers

Developing highly directional nanoscale lasers in TMDC monolayers coupled to dielectric and plasmonic metasurfaces is crucial for on-chip photonic circuits. In this context, the TMDC lasers demonstrated by Barth et al. [106] and Rong et al. [108], which satisfy all established criteria for laser light emission according to the assessment of López-Carrasco and Guimarães [107], provide a firm foundation for this concept. Barth et al. achieved lasing at room temperature in a monolayer ${\mathrm{WS}}_{2}$ that had been grown by CVD and had been coupled to a dual-resonance dielectric metasurface, while Rong et al. described a room-temperature ${\mathrm{WS}}_{2}$ spin–valley Rashba laser (λ=618.2 nm, Q≈5600) based on spin-split high-*Q* BIC modes, thereby allowing for directional, spin–momentum-locked $\sigma_{\pm }$ emission. Based on these results, dielectric quasi-BIC metasurfaces that function in the visible spectrum could offer the high Q value and momentum-selective out-coupling needed for low-threshold directional lasing. Additionally, plasmonic platforms, collective SLRs with high-quality fabrication, could achieve higher Q factor values and narrower angular emission when coupled with TMDC monolayers. Such architectures could be extended from visible-emitting ${\mathrm{WS}}_{2}$ and ${\mathrm{WSe}}_{2}$ toward longer-wavelength monolayers such as ${\mathrm{MoTe}}_{2}$, for which room-temperature continuous-wave laser-like emission has been reported in a silicon nanobeam cavity [105], albeit without satisfying the quantum threshold condition [107]. Although monolayer ${\mathrm{MoTe}}_{2}$ has also been integrated with a plasmonic quasi-BIC metasurface, lasing from such a metasurface geometry has not yet, to our knowledge, been demonstrated. In the end, combining a TMDC monolayer with high-*Q*, momentum-engineered metasurface modes can achieve compact surface-emitting lasers, with the emission wavelength, polarisation, and far-field direction directly controlled by the metasurface, offering a possible platform for highly directional nanoscale light sources.

3. Polariton–polariton interaction in multilayer metasurface–TMDCs systems

Exciton polaritons have a low effective mass of order $10^{-5}$–$10^{-4}\;m_{e}$ from their photonic component and a Coulomb-mediated nonlinearity from their excitonic component, so they propagate over micron distances at high speed while still interacting strongly with one another [119]. This combination is what makes them attractive for compact, ultrafast nanophotonic devices [120]. As a result, conventional multistack lithography can fabricate such a system. First, EBL patterns a dielectric metasurface supporting a high-*Q*-factor qBIC; a TMDC monolayer can then be transferred onto the metasurface, separated by a thin dielectric spacer, placing the TMDC monolayer at the maximum field. Because of the large exciton oscillator strength and binding energy of TMDCs, this stack enters the strong coupling regime at room temperature (see Section 4.2). A multilayer TMDC film of thickness ∼50 nm is subsequently deposited on top. Because of its high refractive index, residual A-exciton oscillator strength, and qBIC design, this layer supports self-hybridised polaritons without an external cavity. Hence, the structure hosts two spatially separated polariton populations whose evanescent tails overlap through the spacer [109]. The resulting polariton–polariton coupling provides access to an interaction strength tunable through spacer thickness and detuning, which is crucial for low-threshold lasing [121] and quantum light generation via polariton blockade [18].

4. Prospects for Integrated Metasurface–TMDC Devices

Beyond the light emission applications discussed above, metasurface–TMDC hybrids offer opportunities for multifunctional and integrated photonic devices. Advances in high-Q resonance engineering, particularly through quasi-BICs and SLRs, could enhance light–matter interaction and enable electrically controlled optical modulation and switching [122]. Together with the potential for quantum state manipulation [123], these capabilities open up opportunities for reconfigurable photonic circuits, integrated quantum technologies, and nanoscale optical sensing. Realising these functionalities will require improved fabrication precision, reduced optical losses, and the scalable integration of TMDC monolayers with metasurface platforms.

## Figures and Tables

**Table 2:** Comparative analysis of representative dielectric and plasmonic metasurface–TMDC monolayer systems exhibiting strong light–matter coupling and their implications for light emission applications.

Metasurface	TMDC	Resonant Mode	*Q*	ℏΩ (meV)	Condition	Light Emission Functionality	Ref.
**Experimental: plasmonic metasurfaces**							
Au nanodisk lattice	${\mathrm{WSe}}_{2}$	SLR	32	44 ±5	4 K	Directional, narrow-angle, and strongly linearly polarised lower-polariton emission governed by the plasmonic lattice.	[92]
Plasmonic quasi-BIC grating	${\mathrm{WS}}_{2}$	Plasmonic quasi-BIC	∼56	93	RT	Exciton–polariton formation and LPB emission, although metal loss limits nonlinear polariton behaviour.	[14]
**Experimental: dielectric metasurfaces**							
SiN metasurface	${\mathrm{WSe}}_{2}$	GMR	∼143	18	22 K	Directional polariton emission with lithographically tunable polariton dispersion and far-field radiation pattern.	[81]
**Numerical: plasmonic metasurfaces**							
Au metasurface	${\mathrm{MoSe}}_{2}$	Catenary-like localised gap plasmon mode	–	133.61	RT (numerical)	Strong exciton–plasmon coupling enabled by nanoscale catenary field confinement and small effective mode volume.	[15]
Ag metasurface	${\mathrm{MoS}}_{2}$	Catenary-like localised gap plasmon mode	–	117.27	RT (numerical)	Large plexcitonic splitting and enhanced spontaneous emission enabled by strong gap field confinement and an ultrasmall optical mode volume.	[50]
**Numerical: dielectric metasurfaces**							
${\mathrm{Si}}_{3}$$N_{4}$ BIC metasurface	${\mathrm{WSe}}_{2}$	Magnetic dipole quasi-BIC	–	38	RT (numerical)	Strong coupling enhanced by optimising the monolayer position, resonator *Q* factor, and metasurface thickness.	[10]
${\mathrm{Si}}_{3}$$N_{4}$ BIC metasurface	${\mathrm{WS}}_{2}$	Magnetic dipole quasi-BIC	–	65	RT (numerical)	Large Rabi splitting by the dielectric QBIC design.	[10]
LN metasurface	${\mathrm{MoS}}_{2}$	GMR/Fano-shaped photonic resonance	∼${110\;}^{*}$	52.76	RT (numerical)	Strong exciton–photon coupling enabled by electromagnetic field confinement within the LN metasurface and localised hotspots in the narrow air gap.	[3]
LN metasurface	${\mathrm{WSe}}_{2}$	GMR/Fano-shaped photonic resonance	≲${110\;}^{*}$	40.35	RT (numerical)	Strong coupling enabled by the high-*Q* dielectric resonance and enhanced field overlap with the monolayer positioned near the electric field maximum.	[3]

Note: *Q* denotes the quality factor of the relevant uncoupled metasurface resonance, while ℏΩ denotes the experimentally measured or theoretically calculated Rabi splitting. A dash indicates that a directly comparable *Q* factor was not explicitly reported for the corresponding resonance.

**Table 3:** Summary of experimental demonstrations and numerical modelings of single-photon emission from monolayers integrated with plasmonic and dielectric metasurfaces/nanostructures.

Metasurface/Nanostructure	1L-TMDCs	Temp.	Coupling Regime	Achievement Including $g^{\left(2\right)}\left(0\right)$	Ref.
**Experimental demonstrations**					
${\mathrm{SiO}}_{2}$ dielectric nanopillar array	${\mathrm{WSe}}_{2}$	3.5 K	Weak; no engineered optical resonance	Deterministic strain-localised SPE array; $g^{\left(2\right)}\left(0\right)=0.07\pm 0.04$	[100]
Au nanocube-on-mirror gap plasmon cavity array	${\mathrm{WSe}}_{2}$	3.8 K	Weak (Purcell regime)	Deterministic plasmon-coupled SPE; $g^{\left(2\right)}\left(0\right)=0.15\pm 0.03$ after cavity coupling (average $g^{\left(2\right)}\left(0\right)=0.21$); maximum $F_{P}=551$	[5]
**Experimental demonstrations**					
${\mathrm{SiO}}_{2}$ nanopillar array with site-selective e-beam defect engineering	${\mathrm{WSe}}_{2}$	5–150 K	Weak; no engineered optical resonance	Site-controlled strain- and defect-localised SPE; $g^{\left(2\right)}\left(0\right)=0.27\pm 0.05$ at 150 K	[95]
${\mathrm{Si}}_{3}$$N_{4}$ dielectric nanogap rod array	${\mathrm{WSe}}_{2}$	4 K	Weak/no resonant coupling	Deterministic position- and polarisation-controlled SPE; $g^{\left(2\right)}\left(0\right)=0.108\pm 0.041$ (minimum 0.045±0.030); 83% emitter yield	[101]
qBIC monocrystalline Si nanoantenna array	${\mathrm{WSe}}_{2}$	4 K	Weak coupling (Purcell regime)	$g^{\left(2\right)}\left(0\right)=0.19\pm 0.02$; effective Purcell factor ∼10; polarisation-selective and directional emission (∼±12°)	[98]
**Numerical modelings**					
Au EQ plasmonic metasurface	${\mathrm{WSe}}_{2}$	300 K	Strong coupling	UnPB; $g^{\left(2\right)}\left(0\right)=1.64\times 10^{-4}$ at $\Omega_{p}=0.5$ meV, demonstrating near-unity single-photon purity	[18]
Au plasmonic metasurface	${\mathrm{WSe}}_{2}$	300 K	Weak coupling (Purcell regime)	UnPB; $g^{\left(2\right)}\left(0\right)=0.0012$ under optimised weak driving; $F_{P}=1310$ at a 10 nm emitter–cavity separation	[18]

Note. Since very few studies have reported single-photon emission from TMDC monolayers strongly coupled to metasurfaces, weak coupling systems, including nanostructured cavities, are also included. Studies with experimental emission measurements and supporting simulations are grouped under experimental demonstrations.

**Table 4:** Room-temperature lasing and stimulated emission in metasurface–TMDC monolayer systems. A check mark (✓) indicates that the corresponding lasing criterion was experimentally demonstrated, whereas × indicates that it was not demonstrated or was not reported. “SE” denotes stimulated emission. The classification distinguishes comprehensive lasing demonstrations from laser-like stimulated emission for which the available evidence is insufficient to establish all lasing criteria.

TMDC	Metasurface	*Q*- Factor	Threshold	Linewidth Narrowing	Coherence	Output Power	Directionality	Classification	Ref.
${\mathrm{WS}}_{2}$ (1L)	${\mathrm{Si}}_{3}$$N_{4}$ dielectric metasurface	∼3000	0.2–$0.5\;W\;{\mathrm{cm}}^{-2}$	✓	✓	✓	✓	Lasing	[106]
${\mathrm{WS}}_{2}$ (1L)	Photonic spin lattice (BIC platform)	∼5600	$2.0\;\mathrm{kW}\;{\mathrm{cm}}^{-2}$	✓	✓	–	✓	Spin–valley lasing	[108]
${\mathrm{WS}}_{2}$ (1L)	Au plasmonic metagrating	∼54	$2.59\;\mathrm{MW}\;{\mathrm{cm}}^{-2}$	✓	×	×	✓	Directional SE	[24]

## Data Availability

No new data were created in this study. Data sharing is not applicable to this article.

## Abbreviations

The abbreviations listed below are used throughout this manuscript:

2D	Two-Dimensional
Au	Gold
BIC	Bound State in the Continuum
CPB	Conventional Photon Blockade
EBL	Electron Beam Lithography
GMR	Guided-Mode Resonance
hBN	Hexagonal Boron Nitride
LP	Lower Polariton
MIR	Mid-Infrared
${\mathrm{MoS}}_{2}$	Molybdenum Disulfide
${\mathrm{MoSe}}_{2}$	Molybdenum Diselenide
NIR	Near-Infrared
Fp	Purcell Factor
Q factor	Quality Factor
qBIC	Quasi-Bound State in the Continuum
${\mathrm{Si}}_{3}$ $N_{4}$	Silicon Nitride
${\mathrm{SiO}}_{2}$	Silicon Dioxide
SLR	Surface Lattice Resonance
TMDC	Transition Metal Dichalcogenide
UP	Upper Polariton
${\mathrm{WS}}_{2}$	Tungsten Disulfide
${\mathrm{WSe}}_{2}$	Tungsten Diselenide
UnPB	Unconventional Photon Blockade
VCSEL	Vertical Cavity Surface-Emitting Laser
